# Mathematical modelling for health systems research: a systematic review of system dynamics and agent-based models

**DOI:** 10.1186/s12913-019-4627-7

**Published:** 2019-11-19

**Authors:** Rachel Cassidy, Neha S. Singh, Pierre-Raphaël Schiratti, Agnes Semwanga, Peter Binyaruka, Nkenda Sachingongu, Chitalu Miriam Chama-Chiliba, Zaid Chalabi, Josephine Borghi, Karl Blanchet

**Affiliations:** 1 Department of Global Health and Development, London School of Hygiene and Tropical Medicine, 15-17 Tavistock Place, London, WC1H 9SH UK; 2 Department of Mathematics, University College London, London, UK; 3 Sia Partners UK, London, UK; 4 Information Systems Department, College of Computing and Information Sciences, Makerere University, P.O. Box 7062, Kampala, Uganda; 5 Ifakara Health Institute, PO Box 78373, Dar es Salaam, Tanzania; 6 Department of Gender Studies, School of Humanities and Social Sciences, University of Zambia, 10101 Lusaka, Zambia; 7 Economic and Business Research Programme, University of Zambia, Institute of Economic and Social Research, P O Box 30900, 10101 Lusaka, Zambia; 8 Department of Public Health, Environments and Society, London School of Hygiene and Tropical, London, UK

**Keywords:** System dynamics, Agent-based, Hybrid, Health systems, Systematic review, Modelling

## Abstract

**Background:**

Mathematical modelling has been a vital research tool for exploring complex systems, most recently to aid understanding of health system functioning and optimisation. System dynamics models (SDM) and agent-based models (ABM) are two popular complementary methods, used to simulate macro- and micro-level health system behaviour. This systematic review aims to collate, compare and summarise the application of both methods in this field and to identify common healthcare settings and problems that have been modelled using SDM and ABM.

**Methods:**

We searched MEDLINE, EMBASE, Cochrane Library, MathSciNet, ACM Digital Library, HMIC, Econlit and Global Health databases to identify literature for this review. We described papers meeting the inclusion criteria using descriptive statistics and narrative synthesis, and made comparisons between the identified SDM and ABM literature.

**Results:**

We identified 28 papers using SDM methods and 11 papers using ABM methods, one of which used hybrid SDM-ABM to simulate health system behaviour. The majority of SDM, ABM and hybrid modelling papers simulated health systems based in high income countries. Emergency and acute care, and elderly care and long-term care services were the most frequently simulated health system settings, modelling the impact of health policies and interventions such as those targeting stretched and under resourced healthcare services, patient length of stay in healthcare facilities and undesirable patient outcomes.

**Conclusions:**

Future work should now turn to modelling health systems in low- and middle-income countries to aid our understanding of health system functioning in these settings and allow stakeholders and researchers to assess the impact of policies or interventions before implementation. Hybrid modelling of health systems is still relatively novel but with increasing software developments and a growing demand to account for both complex system feedback and heterogeneous behaviour exhibited by those who access or deliver healthcare, we expect a boost in their use to model health systems.

## Introduction

Health systems are complex adaptive systems [1]. As such, they are characterised by extraordinary complexity in relationships among highly heterogeneous groups of stakeholders and the processes they create [2]. Systems phenomena of massive interdependencies, self-organising and emergent behaviour, non-linearity, time lags, feedback loops, path dependence and tipping points make health system behaviour difficult and sometimes impossible to predict or manage [3]. Conventional reductionist approaches using epidemiological and implementation research methods are inadequate for tackling the problems health systems pose [4]. It is increasingly recognised that health systems and policy research need a special set of approaches, methods and tools that derive from systems thinking perspectives [5]. Health systems encompass a many tiered system providing services to local, district and national populations, from community health centres to tertiary hospitals. Attempting to evaluate the performance of such a multi-faceted organisation presents a daunting task. Mathematical modelling, capable of simulating the behaviour of complex systems, is therefore a vital research tool to aid our understanding of health system functioning and optimisation.

### System dynamics model (SDM)

System dynamics models (SDM) and agent-based models (ABM) are the two most popular mathematical modelling methods for evaluating complex systems; while SDM are used to study macro-level system behaviour such as the movement of resources or quantities in a system over time, ABM capture micro-level system behaviour, such as human decision-making and heterogeneous interactions between humans.

While use of SDM began in business management [6, 7] it now has wide spread application from engineering to economics, from environmental science to waste and recycling research [8–13]. A SDM simulates the movement of entities in a system, using differential equations to model over time changes to system state variables. A stock and flow diagram can be used to provide a visual representation of a SDM, describing the relationships between system variables using stocks, rates and influencing factors. The diagram can be interpreted as mimicking the flow of water in and out of a bath tub [7]; the rates control how much ‘water’ (some quantifiable entity, resource) can leave or enter a ‘bath tub’ (a stock, system variable) which changes over time depending on what constraints or conditions (e.g. environmental or operational) are placed on the system. Often before the formulation of a stock and flow diagram, a causal loop diagram is constructed which can be thought of as a ‘mental model’ of the system [14], representing key dynamic hypotheses.

### Agent-based model (ABM)

Unlike SDM, ABM is a ground-up representation of a system, simulating the changing states of individual ‘agents’ in a system rather than the broad entities or aggregate behaviour modelled in SDM. Aggregate system behaviour can however be inferred from ABM. Use of ABM to model system behaviour has been trans-disciplinary, with application in economics to ecology, from social sciences to engineering [15–19]. There can be multiple types of agent modelled, each assigned their own characteristics and pattern of behaviour [20, 21]. Agents can learn from their own experiences, make decisions and perform actions based on set rules (e.g. heuristics), informed by their interactions with other agents, their own assigned attributes or based on their interaction with the modelled environment [22]. The interactions between agents can result in three levels of communication between agents; one-to-one communication between agents, one-to-many communication between agents and one-to-location communication where an agent can influence other agents contained in a particular location [22].

### Why use SDM and ABM to model health systems?

ABM and SDM, with their ability to simulate micro- and macro-level behaviour, are complementary instruments for examining the mechanisms in complex systems and are being recognised as crucial tools for exploratory analysis. Their use in mapping health systems, for example, has steadily risen over the last three decades. ABM is well-suited to explore systems with dynamic patient or health worker activity, a limitation of other differential equation or event-based simulation tools [23–25]. Unlike discrete-event simulation (DES) for example, which simulates a queue of events and agent attributes over time [26], the agents modelled in ABM are decision makers rather than passive individuals. Closer to the true system modelled, ABM can also incorporate ongoing learning from events whereby patients can be influenced by their interactions with other patients or health workers and by their own personal experience with the health system [21]. SDM has also been identified as a useful tool for simulating feedback and activity across the care continuum [27–30] and is highly adept at capturing changes to the system over time [31]. This is not possible with certain ‘snapshot in time’ modelling approaches such as DES [32]. SDM is best implemented where the aim of the simulation is to examine aggregate flows, trends and sub-system behaviour as opposed to intricate individual flows of activity which are more suited to ABM or DES [33].

There are also models that can accommodate two or more types of simulation, known as hybrid models. Hybrid models produce results closer to true system behaviour by drawing on the strengths of one or more modelling methods while reducing the limitations associated with using a single simulation type [27]. The activity captured in such models emulates the individual variability of patients and health professionals while retaining the complex, aggregate behaviour exhibited in health systems.

Health scientists and policy makers alike have recognised the potential of using SDM and ABM to model all aspects of health systems in support of decision making from emergency department (ED) optimisation [34] to policies that support prevention or health promotion [35]. Before implementing or evaluating costly health policy interventions or health service re-structuring in the real world, modelling provides a relatively risk-free and low budget method of examining the likely impact of potential health system policy changes. They allow the simulation of ‘what if’ scenarios to optimise an intervention [36]. They can help identify sensitive parameters in the system that can impede the success of initiatives and point to possible spill-over effects of these initiatives to other departments, health workers or patients. Perhaps most important of all, these modelling methods allow researchers to produce simulations, results and a graphical-user interface in relation to alternative policy options that are communicable to stakeholders in the health system [37], those responsible for implementing system-wide initiatives and changes.

### Study aim and objectives

Given the increasing amount of literature in this field, the main aim of the study was to examine and describe the use of SDM and ABM to model health systems. The specific objectives were as follows: (1) Determine the geographical, and healthcare settings in which these methods have been used (2) Identify the purpose of the research, particularly the health policies or interventions tested (3) Evaluate the limitations of these methods and study validation, and (4) Compare the use of SDM and ABM in health system research.

Although microsimulation, DES and Markov models have been widely used in disease health modelling and health economic evaluation, our aim in this study was to review the literature on mathematical methods which are used to model complex dynamic systems, SDM and ABM. These models represent two tenants of modelling: macroscopic (top-level) and microscopic (individual-level) approaches. Although microsimulation and DES are individual-based models like ABM, individuals in ABM are “active agents” i.e. decision-makers rather than “passive agents” which are the norm in microsimulation and DES models. Unlike Markov models which are essentially one-dimensional, unidirectional and linear, SDM are multi-dimensional, nonlinear with feedback mechanisms. We have therefore focussed our review on SDM and ABM because they are better suited to characterise the complexity of health systems. This study reviews the literature on the use of SDM and ABM in modelling health systems, and identifies and compares the key characteristics of both modelling approaches in unwrapping the complexity of health systems. In identifying and summarising this literature, this review will shed light on the types of health system research questions that these methods can be used to explore, and what they add to more traditional methods of health system research. By providing an over overview of how these models can be used within health system research, this paper is also expected to encourage wider use and uptake of these methods by health system researchers and policy makers.

## Methods

The review was conducted in compliance with the Preferred Reporting Items for Systematic Reviews and Meta-Analysis (PRISMA) statement [38].

### Search strategy and information sources

The literature on ABM and SDM of health systems has not been confined to a single research discipline, making it necessary to widen the systematic review to capture peer-reviewed articles found in mathematical, computing, medicine and health databases. Accordingly, we searched MEDLINE, EMBASE, Cochrane Library, MathSciNet, ACM Digital Library, HMIC, Econlit and Global Health databases for literature. The search of health system literature was narrowed to identify articles that were concerned with modelling facility-based healthcare, services and related healthcare financing agreements which had been excluded or were not the focus of previous reviews [34, 35, 39–41]. The search criteria used for MEDLINE was as follows, with full search terms for each database and search terms used to locate SDM and ABM literature found in Additional file 1:

(health system* OR health care OR healthcare OR health service* OR health polic* OR health facil* OR primary care OR secondary care OR tertiary care OR hospital*).ab,ti. AND (agent-based OR agent based).ab,ti. AND (model*).ab,ti.

In addition, the reference list of papers retained in the final stage of the screening process, and systematic reviews identified in the search, were reviewed for relevant literature.

### Data extraction and synthesis

**Fig. 1:**
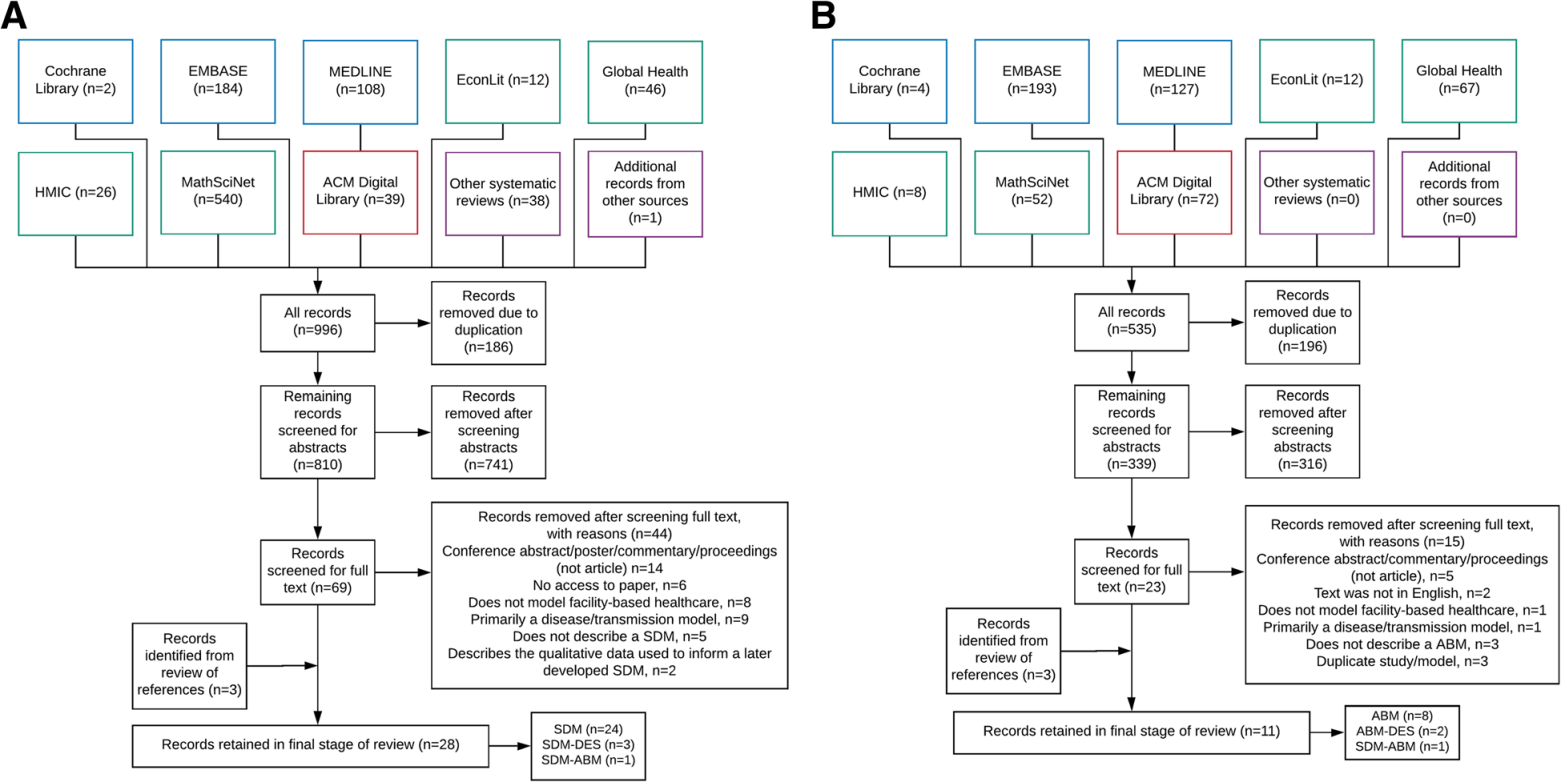
**a** Flow-chart for systematic review of SDMs and **b** ABMs of health systems (Database research discipline is identified by colour; mathematical and computing (red), medicine (blue) and health (green) databases). Adapted from PRISMA [38]

The screening process for the review is given in Fig. 1 (adapted from [38]). All search results were uploaded to Mendeley reference software where duplicate entries were removed. The remaining records were screened using their titles and abstracts, removing entries based on eligibility criteria given in Table 1. Post-abstract review, the full text of remaining articles was screened. Papers retained in final stage of screening were scrutinised, with data imported to Excel based on the following categories; publication date, geographical and healthcare setting modelled, purpose of research in addition to any policies or interventions tested, rationale for modelling method and software platform, validation and limitations of model. The results were synthesised using descriptive statistics and analysis of paper content that were used to answer the objectives.

**Table 1:** Eligibility criteria for review

Criteria	Inclusion	Exclusion
Type of study/model	Studies that describe the development and presentation of SDM or ABM or hybrid model.	Poster presentations, conference abstracts, review papers (reference list reviewed), commentaries, debate papers, papers that describe the qualitative data used to inform a later developed model, papers that only present conceptual SDM or ABM model, papers that present exclusively a DES model or other modelling method.
Setting	Facility-based healthcare or related policies/financing arrangements	Papers that primarily describe a disease/transmission model or delivery of non-facility-based healthcare
Publication date	Up to May 2019	
Language	English	Other languages

**Fig. 2:**
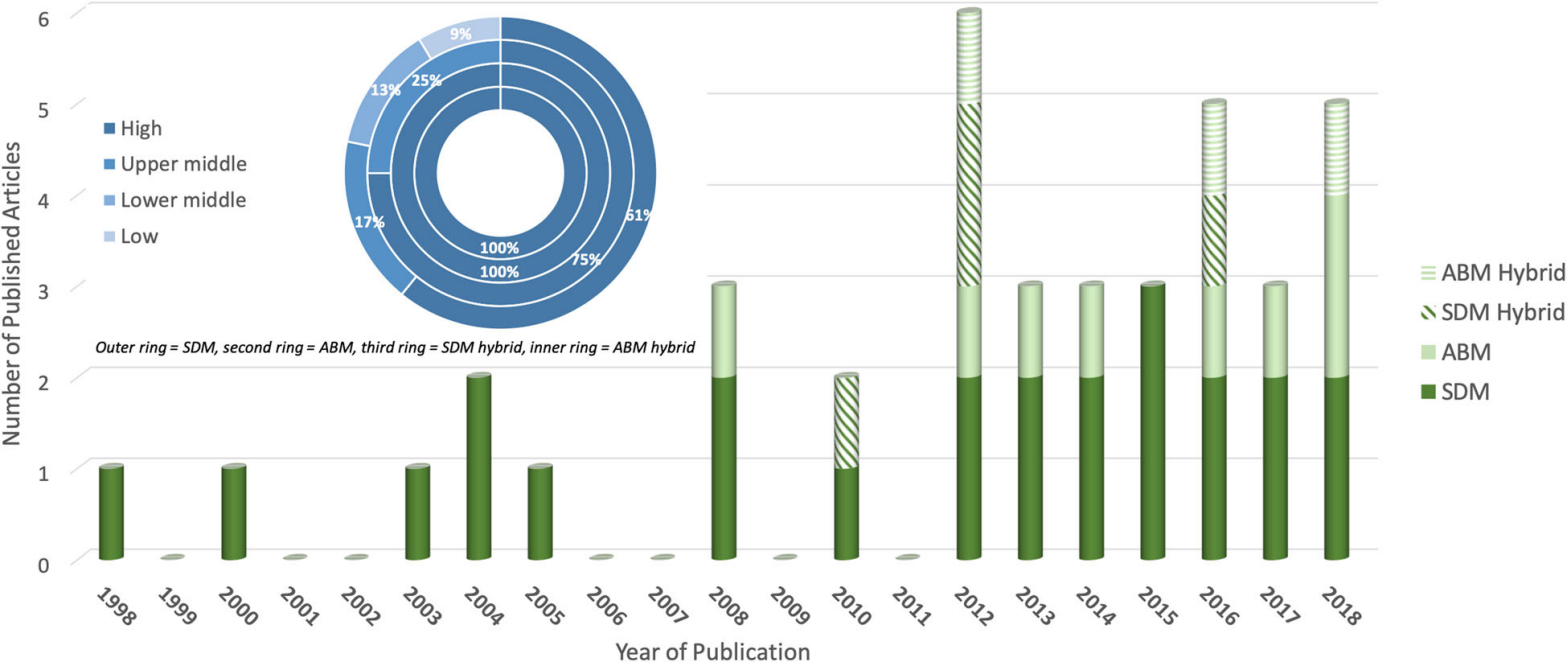
Number of articles in the final review by year of publication and economic classification

The studies were first described by three characteristics: publication date, geographical setting, and what aspect of the health system was modelled and why. These characteristics were chosen for the following reasons. Publication date (Fig. 2) allows us to examine the quantity of SDM and ABM studies over time. Geographical settings (Fig. 2, top) allows us to see which health systems have been studied, as health systems in LMIC are very different from those in developed countries. Studies are classified as modelling health systems in high, upper middle, lower middle and low income countries as classified by The World Bank based on economy, July 2018 [42]. Finally, we examined which aspects of the health system have been modelled and the types of research/policy questions that the models were designed to address, to shed light on the range of potential applications of these models, and also potential gaps in their application to date.

The analysis of paper content was split into three sections; SDM use in health system research (including hybrid SDM-DES), ABM use in health system research (including hybrid ABM-DES) and hybrid SDM-ABM use in health system research. The quality of selected studies will not be presented as our aim was to compare and summarise the application of SDM and ABM in modelling health systems rather than a quality appraisal of studies.

## Results

### Study selection

The search initially yielded 535 citations for ABM and 996 citations for SDM of facility-based healthcare and services (see Fig. 1). Post-full text screening 11 ABM and 28 SDM papers were retained for analysis, six of which utilised hybrid modelling methods. Three of the hybrid modelling papers integrated SDM with DES [43–45], two integrated ABM with DES [24, 46] and one integrated SDM with ABM [47]. A summary table of selected papers is given in Table 2.

**Table 2:** Summary of studies included at full paper review (SDM) and studies included at full paper review (ABM)

Paper/Year/Ref	Purpose	Sector of health system modelled	Key results	Software platform
System dynamics models (SDMs)				
Al-Khatib (2016) [48]	Assess the impact of key factors on the hospital waste management system and compare the future total waste output between private, charitable and government hospitals.	• Model simulates hospital waste management in Nablus, Palestine. • Focus on three different types of hospital (private, charitable and government hospitals).	• The amount of waste generated heavily dependent on the number of beds. • Waste treatment was dependent on staff training and the enforcement of legislation.	• iThink.
Alonge (2017) [30]	Explore effective implementation structure for improving health system performance through pay-for-performance (P4P) initiative.	• The model is a generic representation of the pay for performance initiative in primary health facilities in Afghanistan.	• P4P initiative would likely have a beneficial impact on the volume and quality of health services if correctly implemented. • May prove ineffective if the impact of gaming is not mitigated or if the method for distributing financial rewards are inadequate.	• MATLAB and Simulink.
Ansah (2014) [49]	Assess the impact of different long-term care (LTC) capacity policies on uptake of acute care, demand for and utilisation of LTC services.	• Generic representation of LTC utilisation and resources for care and is not based or set in a particular health facility.	• Proactive adjustment of LTC capacity stemmed the number of acute care visits but required a modest increase in staff. • Movement of health staff (through delayed training or from LTC to the acute care sector) will impede the success of this policy.	• Does not state.
Brailsford (2004) [50]	To determine how emergency and on demand care is currently configured and what policies could alleviate pressure on the health system.	• Entire healthcare system that provides emergency centres etc) in Nottingham, England.	• Significant impact on elective hospital admissions as emergency cases are currently prioritised. • Redirecting certain elderly patients to appropriate services relieved pressure on emergency services.	• STELLA.
Brailsford (2010)^a^ [43]	Investigate how local authorities such as Hampshire County Council (HCC) can improve access to services and support for older people, in particular assess the long-term impact of a new contact centre for patients.	• HCC system for long-term care, including a call centre that older patients can access to receive advice or be directed to appropriate care.	• The number of patients who contact the call centre on a second occasion (having failed to make contact the first time) where the health status of the patient has now deteriorated, fell drastically after the introduction of two additional call handlers.	• SDM is Vensim, DES model is Simul8.
Cepoiu-Martin (2018) [51]	To examine patient transition from home to supportive living (SL) or long term care (LTC) in persons with dementia and discern policy impact on the deficit of nurses and health care assistants.	• The Alberta Continuing Care System comprising of home living, SL or LTC services.	• Introducing benchmarks for hiring nurses and health care assistants in SL and LTC facilities will result initially in a greater deficit of staff but will stabilise the ratio of health professionals to patients in the long term.	• Does not state.
Chaerul (2008) [37]	To determine key factors that impact the management of hospital waste and predict future waste output.	• The model describes hospital waste management in the City of Jakarta, Indonesia.	• Hospital waste disposal is impacted by the reluctance of a densely populated cityto allow further waste to be dumped in landfill sites. • The simulation indicated that existing and new landfill sites will be at full capacity by 2011 and 2020, respectively.	• STELLA.
Ciplak (2012) [52]	To predict future healthcare waste production and optimise the management of healthcare waste.	• Healthcare waste generation from healthcare facilities, the single healthcare waste treatment facility and alternative waste treatment facilities in Istanbul, Turkey.	• Employing stringent waste separation strategies would relieve the pressure on already at capacity waste treatment facility in Istanbul. • Up to 77% of healthcare waste could be diverted to alternative treatment technologies that do not require treatment at the incineration facility.	• Vensim.
De Andrade (2014) [53]	To examine the reasons for delayed ST-segment elevation myocardial infarction (STEMI) treatment and explore interventions that can speed up wait time in primary care facilities.	• A primary care hospital and a Percutaneous Coronary Intervention Centre (PCI) in Brazil.	• It was observed that 50% reduction in waiting time for patients is possible under a combination of interventions targeting ECG transmission and PCI centre team feedback time and patient transfer waiting time.	• Vensim.
Desai (2008) [54]	To forecast demand for older people’s services and explore the future impact of challenges that accompany an ageing population.	• Adult Services Department of Hampshire County Council including 13 different types of care package that can be offered by the funding and assessment body.	• Providing care packages only to critical patients reduced the overall number of patients receiving acute care. • Savings can be made by increasing the number of unqualified care workers which can be fed back into care funding.	• STELLA.
Djanatliev (2012)^b^ [47]	Presenting the functionality of the Prospective Health Technology Assessment (ProHTA) tool, which can simulate the impact of optimised technology prospectively before physical development.	• Mobile Stroke Unit (MSU) case study was simulated for Berlin, includes a generic hospital with emergency services where patients are taken by the MSU.	• In the simulation implementing MSU, 18.2% of patients received thrombolysis treatment compared with 10.6% in the simulation without MSU. • Fewer patients were also found to have developed severe disability in the simulation with MSU as a consequence of faster implemented treatment, reducing the long term costs for rehabilitation and care.	• AnyLogic.
Eleyan (2013) [55]	To predict general and medical waste generation for a complex hospital waste management system.	• Model simulates hospital waste management in three hospitals based in Jenin, Palestine.	• Increases in the amount of hospital waste are consistent with bed occupancy. Over the next 20 years, the total amount of waste generated will rise as will the total cost of treating hazardous waste.	• iThink.
Esensoy (2018) [28]	Transformation of stroke care to implement best practice.	• The model describes six sectors of Ontario health care system and the patient flow between them.	• When stroke best practice policy has been implemented (compared to the base case scenario), there is a reduction in length of stay across all sectors. • A reduction in bed utilisation was also observed with a 10 and 11.1% reduction in acute care and rehab sectors, respectively.	• Vensim.
Ghaffarzad. (2013) [32]	To explore physician decision making behind scheduled caesarean delivery (CD), unplanned CD and vaginal delivery (VD) and examine factors that influence procedure variation.	• The model does not reflect a particular hospital but is parameterised using patient information from hospital discharge databases in Florida.	• The biggest impact on physician delivery decision is from the delayed effect of colleague past experience. • Turning off all learning experiences reduces physician delivery variation for scheduled CD delivery from 6.5 to 4.7%.	• Vensim.
Lane (1998) [56]	Explore the factors that lead to delays in Accident and Emergency Departments (A&E) and to elective admissions.	• A&E department at major inner-London teaching hospital coded in the study as ‘St Dane’s’.	• Reduction in bed numbers increases emergency admission waiting times and delays and cancellations to elective surgery admissions. • Increases in demand push the system to breaking point, with patients waiting hours to be admitted and health workers at full capacity.	• Does not state.
Lane (2000) [57]	The model depicts the performance of Accident and Emergency (A&E) at acute hospitals, investigating the sensitivity of waiting times to hospital bed numbers.	• A&E department at Inner-London teaching hospital coded in the study as ‘St Dane’s’.	• Reducing bed capacity increased the % of elective cancellations, negating the impact on other performance measures. • Deterioration of services is not attributed to lack of bed capacity but insufficient provision of A&E doctors who reach 100% utilisation.	• iThink.
Lattimer (2004) [36]	To evaluate ‘front door’ services of local emergency and urgent care facilities and test proposals for system change.	• Entire healthcare system that provides emergency or on demand care (GP, NHS Direct, Walk in centres etc) in Nottingham.	• Reducing emergency admissions from GP by 4% showed successive reduction in occupancy levels in A&E. • Interventions to lower admissions of patients over 60 resulted in a 1% reduction per annum in bed occupancy over 5 years.	• STELLA.
Mahmoudia. (2017) [58]	To explore the intended and unintended consequences of Intensive Care Unit (ICU) resource and bed management policies on patient mortality, emergency departments (ED) and general wards.	• Generic model of ICU, ED and general hospital wards.	• Whilst general ward admission control is not as effective at reducing ICU and ED occupancy rates, it outperforms other policies with regards to reducing patient mortality, arguably the more important ICU management performance measure.	• Does not state.
Meker (2015) [59]	To describe performance-based payment systems (PBPS) in second-step public hospitals and the impact on process measures in hospitals.	• Second-step public hospitals in Turkey.	• With reduced performance payments, physicians move to the private sector decreasing staff levels, reducing time spent with patients leading to a dramatic decrease of correct diagnosis and treatment.	• Does not state.
Mielczarek (2016)^a^ [44]	To estimate the future demand for healthcare from patients with cardiac disease.	• Future demand for cardiac disease care in Wroclaw Region, Poland.	• Older population (over 60) will generate increasing demands for care, specifically the growth of cardiac patients was observed as more intense in men than women (increases of 34.4 and 30.15% respectively).	• Does not state.
Rashwan (2015) [31]	To explore the flow of elderly patients through the Irish healthcare system and anticipate the growing demand for services over the next 5 years.	• Generic emergency care facility in Ireland and six possible discharge locations.	• Under increasing demand, a combination of all three policies was necessary to significantly reduce elderly frail patients’ length of stay in acute hospitals and reduce delayed discharge numbers.	• Does not state.
Semwanga (2016) [60]	To capture the dynamics of the Ugandan health system and evaluate what impact interventions might have on neonatal care.	• Does not focus on one type of health facility but incorporates different services and levels of care offered to this group.	• Integrating community health education, free delivery kits and motorcycle coupons has the biggest impact on reducing neonatal death. • Interventions targeting socioeconomic status had a greater impact on reducing neonatal mortality than those targeting service delivery.	• STELLA.
Taylor (2005) [33]	To examine the impact of shifting cardiac catheterization (CC) services from tertiary to secondary level for low risk investigations and explore how improvements could be made to services.	• The CC service pathways at two English district general hospitals, referred to using the pseudonyms ‘Veinbridge Hospital’ and ‘Ribsley Hospital’.	• Significant and stable improvements in service (reducing waiting list time and overall costs of service) were achieved with the implementation of strict referral guidelines for patients.	• STELLA.
Walker (2003) [61]	To model patient flow from feeder hospitals to a sub acute extended care hospital to show the impact of local rules used by the medical registrar (medical admitting officer).	• A single extended care facility in Victoria (Australia) and patient flow from feeder hospitals.	• Using the local rule, the cost of care exceeds the budget by 6%. Without the local rule, costs were 3% under budget. ﻿ • The unprioritized list maintains waiting lists at a level that effectively short-circuits the feeder hospital second local rule of moving high acuity patients on to the wait list of the sub-acute hospital.	• iThink.
Wong (2010) [62]	To evaluate if smoothing the number of discharges over the week relieves the pressure on emergency departments (ED).	• Model describes a general internal medicine (GIM) program at a single tertiary care teaching hospital in Toronto, Canada.	• Both scenarios for ‘smoothed average case’ were similar, resulting in reduction of GIM in ED by 27% and GIM in ED length of stay by 31%. • For ‘every day is a week day case’, larger reductions observed.	• Vensim.
Worni (2012) [63]	To estimate what impact a policy to deny reimbursement of total knee arthroplasty (TKA) patient fees will have on venous thromboembolism (VTE) rates and any unintentional consequences.	• The model simulates all patients (9.7 million) in the US who have symptomatic osteoarthritis, over 65 and have Medicare insurance.	• Model output indicates new policy will result in 3-fold decrease in VTE rates. Fraction of those (in simulation with new policy) with bleeding complications is 6-fold higher and 6-fold more patients ineligible for TKA per year.	• Vensim.
Yu (2015) [64]	To explore the driving factors for a high proportion of patients in China not seeking medical care (also known as potential medical demand) and examine possible interventions.	• Three main sub-systems; medical demand of patients, outpatients in hospitals and outpatients in community health systems (CHS). It does not describe a specific hospital or CHS.	• An increase in the number of CHS and decrease in the number of hospitals was found to induce the biggest decrease in the number of patients not seeking care. • Varying the price of outpatient care in hospitals and CHS had minimal impact on increasing the number of patients who seek care.	• Vensim.
Zulkepli (2012)^a^ [45]	Present a case study using hybrid modelling (SDM-DES), explore patient flow in an integrated care system (IC) and the impact of patient admission on health professional stress level.	• Three main sub-systems; patient flow through critical care facility, patient flow through intermediate care assessment and motivation and stress levels of health professionals.	• Due to high demand of intermediate care services but limited spaces bed blocking may occur, with an increase in patient admissions leading to an increase to health professional stress level.	• SDM is Vensim, DES model is Simul8.
Agent-based models (ABMs)				
Alibrahim (2018) [23]	To explore the effect of patient choice on the healthcare market, specifically providers that form accountable care organisations (ACO).	• A generalised simulation of patient (Medicare beneficiary, over 65 years old who has or can develop congestive heart failure) choice of medical provider (hospital or primary care physician facility) in the United States.	• Where providers were allowed to opt out of ACO network, they were able to optimise their own profits by not implementing a disease management programme - this led to a reduction in the overall quality of care, driving patients to attend alterative care facilities reducing the utilisation of that facility.	• AnyLogic.
Djanatliev (2012)^b^ [47]	Presenting the functionality of the Prospective Health Technology Assessment (ProHTA) tool, which can simulate the impact of optimised technology prospectively before physical development.	• Mobile Stroke Unit (MSU) case study was simulated for Berlin, includes a generic hospital with emergency services where patients are taken by the MSU.	• In the simulation implementing MSU, 18.2% of patients received thrombolysis treatment compared with 10.6% in the simulation without MSU. • Fewer patients were also found to have developed severe disability in the simulation with MSU as a consequence of faster implemented treatment, reducing the long term costs for rehabilitation and care.	• AnyLogic.
Einzinger (2013) [65]	To create a tool capable of comparing reimbursement schemes in outpatient care.	• Compared different reimbursement schemes for Austrian outpatient health sector simulating the vast majority of health insured persons in Austria.	• Creation of a tool that can be used to compare health care reimbursement schemes in Austria.	• AnyLogic.
Hutzsch. (2008) [66]	To determine which mix of patients should be admitted to specialised hospitals to optimise resource utility and to consider the impact of unplanned patient arrivals on this process.	• Cardiothoracic surgery (CTS) and intensive care unit (ICU) at Catharina Hospital Eindhoven (CHE) in the Netherlands. CTS and ICU are broken down into their respective units such as the high care unit of CTS etc.	• An additional ward bed on the CTS ward decreased the frequency of sending pre- and post- operative admissions to other wards by a factor of 3 with minimal cost. • The brute force optimiser indicated that the number of IC high care beds should be increased and number of IC beds decreased to gain optimum throughput of patients in simulation.	• Java.
Huynh (2012) [20]	To assess the impact of redesigning medication administration process (MAP) workflow for registered nurses to improve medication administration safety.	• A local (anonymous) medical centre where nurses are administering medication to patients.	• Implementing a protocol for the order of MAP tasks to be performed improved the amount of time spent performing tasks. • When registered nurses performed tasks in the most frequently observed order (in the pilot study) this improved MAP task times.	• Netlogo.
Kittipitta. (2016)^c^ [24]	To examine patient flow in an outpatient clinic of an orthopedic department and explore interventions that can improve clinical services to reduce patient waiting times.	• Orthopedic department at unidentified community hospital.	• Average waiting time for outpatient appointments fell by 32.03% under the new management policy.	• AnyLogic.
Liu (2014) [21]	To develop a tool that can be used as a decision support system for managers of emergency departments (ED) to assess risk, allocation of resources and identify weakness in emergency care service.	• ED at Hospital of Sabadell (University tertiary level hospital in Barcelona, Spain). The Department is split into sections A (critical patients) and B (least critical patients).	• A tool that can be used simulate the behaviour of agents in ED.	• Netlogo.
Liu (2016) [25]	To explore how accountable care organisations (ACO) can impact payers, healthcare providers and patients under a shared savings payment model for congestive heart failure (CHF) and achieve optimal outcomes.	• A generalised simulation of patients (Medicare beneficiary, over 65 years old who has or can develop congestive heart failure) seeking care (hospital or primary care physician facility) in Unites States.	• Quality orientated providers yielded higher financial returns to the payer agent (which were then shared between providers) than those that were profit-orientated.	• AnyLogic.
Viana (2018)^c^ [46]	To examine and improve patient flow through a pregnancy outpatient clinic in light of the uncertainty in demand for services from overdue patients.	• Overdue pregnancy outpatient clinic, pregnancy clinic and postnatal clinic at Akershus University Hospital, Norway.	• As expected increasing the number of midwives in the clinic reduces resource utilisation but combined with an increase in demand led to an increase in doctor utilisation. • Midwives act as a buffer (or bottleneck) to patients seeing doctors.	• AnyLogic.
Yousefi (2017) [67]	To apply group decision-making techniques for emergency department (ED) resource allocation and determine whether this approach improves performance indicators.	• A generic ED informed from the literature.	• Group-decision making between agents in the ED resulted in on average a 12.7% decrease in total waiting time and 14.4% decrease in the number of patients who left without being seen.	• Netlogo.
Yousefi (2018) [22]	To examine the behaviour of patients who leave public hospital emergency departments (ED) without being seen and the impact of preventative policies.	• ED at Hospital Risoleta Tolentino Neves, a tertiary hospital in Minas Gerais, Brazil.	• After applying preventative policies, average 42.14% reduction in the number of patients leaving without being seen in the ED and average 6.05% reduction in patient length of stay in ED was observed, with most effective policy to fast-track less critical patients after triage.	• NetLogo .

Note: ^a^Articles implemented SDM-DES hybrid modelling

^b^Articles implemented SDM-ABM hybrid modelling

^c^Articles implemented ABM-DES hybrid modelling

### Descriptive statistics

#### Publication date

The first SDM paper to model health systems was published in 1998 [56] whilst the first publication [66] utilising ABM came almost a decade later (Fig. 2). We found an increasing trend in publications for both modelling approaches, with 90.9% (10/11) and 71.4% (20/28) of all ABM and SDM articles, respectively, having been published in the last decade. The first hybrid modelling article was published in 2010 [43], using SDM and DES to model the impact of an intervention to aid access to social care services for elderly patients in Hampshire, England.

#### Geographical setting

The proportion of papers that modelled health systems in high, upper middle, lower middle and low income countries is presented in Fig. 2. Eighteen (18/28) papers that employed SDM simulated health systems in high income countries including England [33, 36, 43, 45, 50, 54, 56, 57] and Canada [28, 51, 62]. Four SDM papers simulated upper middle income country health systems, including Turkey [52, 59] and China [64], with a nominal number of papers (5/28) focussing on lower middle or low income countries (West Bank and Gaza [48, 55], Indonesia [37], Afghanistan [30] and Uganda [60]). Almost all ABM papers (9/11) modelled a high income country health system, including the US [20, 23, 25] and Austria [65]. Two (2/11) ABM papers described an upper-middle income based health system (Brazil [22, 67]). All six articles that implemented a hybrid SDM or ABM simulated health systems based in high income countries, including Germany [44] and Poland [47].

#### Healthcare setting and purpose of research

**Fig. 3:**
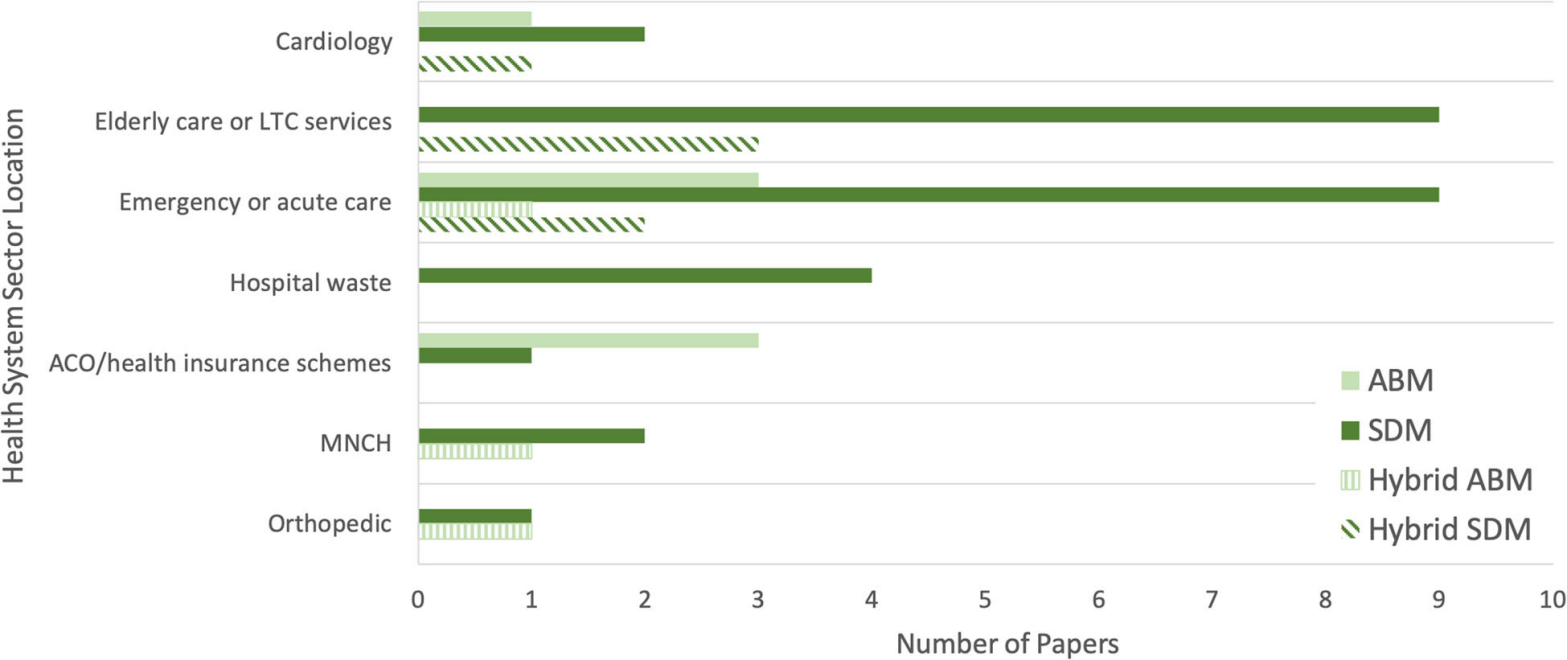
The health system sector locations modelled in the SDM, ABM and hybrid modelling literature. Long-term care (LTC); Accountable care organisation (ACO); Maternal, newborn and child health (MNCH)

The healthcare settings modelled in the SDM, ABM and hybrid simulation papers are presented in Fig. 3. Healthcare settings modelled using SDM included systems that were concerned with delivering emergency or acute care (11/28) [28, 31, 36, 45, 47, 50, 56–58, 61, 62], elderly or long-term care services (LTC)(12/28) [28, 31, 36, 43–45, 49–51, 54, 61, 62] and hospital waste management (4/28) [37, 48, 52, 55]. Twenty of the SDM papers selected in this review assessed the impact of health policy or interventions on the modelled system. Common policy targets included finding robust methods to relieve stretched healthcare services, ward occupancy and patient length of stay [28, 31, 36, 43, 49, 50, 54, 58, 62], reducing the time to patient admission [33, 53, 61], targeting undesirable patient health outcomes [47, 58, 60, 63], optimising performance-based incentive health system policies [30, 59] and reducing the total cost of care [33, 54, 61]. The remaining eight papers explored factors leading to undesirable emergency care system behaviour [56, 57], simulating hospital waste management systems and predicting future waste generation [37, 48, 55], estimating future demand for cardiac care [44], exploring the impact of patient admission on health professionals stress level in an integrated care system [45], and variation in physician decision-making [32].

ABM papers modelled systems focussed on delivering emergency or acute care (4/11) [21, 22, 47, 67] and accountable care organisations (ACO) or health insurance reimbursement schemes (3/11) [23, 25, 65]. Nine of the ABM papers assessed the impact of health policy or interventions on the modelled system. Common policy targets included decreasing the time agents spent performing tasks, waiting for a service or residing in parts of the system [20, 22, 24, 67], reducing undesirable patient outcomes [23, 25, 47, 67], reducing the number of patients who left a health facility without being seen by a physician [22, 67] and optimising resource utility (beds and healthcare staff) [46, 66, 67]. The remaining two papers described simulation tools capable of comparing health insurance reimbursement schemes [65] and assessing risk, allocation of resources and identifying weaknesses in emergency care services [21].

Papers that utilised hybrid simulation, combining the strengths of two modelling approaches to capture detailed individual variability, agent-decision making and patient flow, modelled systems focussed on delivering elderly care or LTC services [43–45] and emergency or acute care [45, 47]. Four of the hybrid simulation papers assessed the impact of policy or intervention on the modelled system. Policy targets included improving access to social support and care services [43], reducing undesirable patient outcomes [47], decreasing patient waiting time to be seen by a physician [24] and improving patient flow through the system by optimising resource allocation [46]. The remaining two papers used hybrid simulation to estimate the future demand for health care from patients with cardiac disease [44] and model patient flow through an integrated care system to estimate impact of patient admission on health care professionals wellbeing [45].

### SDM use in health systems research (including hybrid SDM-DES)

#### Rationale for using model

Gaining a holistic system perspective to facilitate the investigation of delays and bottlenecks in health facility processes, exploring counter-intuitive behaviour and monitoring inter-connected processes between sub-systems was cited frequently as reasons for using SDM to model health systems [28, 36, 37, 48, 56]. SDM was also described as a useful tool for predicting future health system behaviour and demand for care services, essential for health resource and capacity planning [48, 60]. Configuration of the model was not limited by data availability [28, 52, 64] and could integrate data from various sources when required [51].

SDM was described as a tool for health policy exploration and optimising system interventions [33, 36, 51, 54, 58, 64], useful for establishing clinical and financial ramifications on multiple groups (such as patients and health care providers) [63], identifying policy resistance or unintended system consequences [59, 61] and quantifying the impact of change to the health system before real world implementation [62]. The modelling platform also provided health professionals, stakeholders and decision makers with an accessible visual learning environment that enabled engagement with experts necessary for model conception and validation [48, 50, 55, 57]. The model interface could be utilised by decision makers to develop and test alternative policies in a ‘real-world’ framework that strengthened their understanding of system-wide policy impact [31, 49, 58, 61].

SDM-DES hybrid models enabled retention of deterministic and stochastic system variability and preservation of unique and valuable features of both methods [44], capable of describing the flow of entities through a system and rapid insight without the need for large data collection [43], while simulating individual variability and detailed interactions that influence system behaviour [43]. SDM-DES offered dual model functionality [44] vital for simulating human-centric activity [45], reducing the practical limitations that come with using either SDM or DES to model health systems such as attempting to use SDM to model elements which have non-aggregated values (e.g. patient arrival time) [45] which is better suited for DES.

#### Healthcare setting

Sixteen papers that utilised SDM modelled systems that were concerned with the delivery of emergency or acute care, or elderly care or LTC services.

Ten of the reviewed papers primarily modelled sectors of the health system that delivered emergency or acute care^1^^,^^2^. Brailsford et al. [50], Lane et al. [56], Lane et al. [57] and Lattimer et al. [36] simulated the delivery of emergency care in English cities, specifically in Nottingham and London. Brailsford et al. [50] and Lattimer et al. [36] created models that replicated the entire emergency care system for the city of Nottingham, from primary care (i.e. General Practice surgeries) to secondary care (i.e. hospital admissions wards), to aid understanding of how emergency care was delivered and how the system would need to adapt to increasing demand. Lane et al. [56] and Lane et al. [57] modelled the behaviour of an ED in an inner-London teaching hospital, exploring the knock on effects of ED performance to hospital ward occupancy and elective admissions. Esensoy et al. [28] and Wong et al. [62] both modelled emergency care in Canada, Esensoy et al. [28] focussing on six sectors of the Ontario health system that cared for stroke patients while Wong et al. [62] simulated the impact of delayed transfer of General Internal Medicine patients on ED occupancy. Rashwan et al. [31], Walker et al. [61] and Mahmoudian-Dehkordi et al. [58] modelled patient flow through a generic emergency care facility with six possible discharge locations in Ireland, a sub-acute extended care hospital with patient flow from feeder facilities in Australia and an intensive care unit, ED and general wards in a generic facility.

Five of the SDM papers primarily simulated the behaviour of LTC facilities or care services for elderly patients^3^. Ansah et al. [49] modelled the demand and supply of general LTC services in Singapore with specific focus on the need for LTC and acute health care professionals. Desai et al. [54] developed a SDM that investigated future demand of care services for older people in Hampshire, England which simulated patient flow through adult social care services offering 13 different care packages. In modelling complex care service demand, Cepoiu-Martin et al. [51] explored patient flow within the Alberta continuing care system in Canada which offered supportive living and LTC services for patients with dementia. Brailsford et al. [43] used a hybrid SDM-DES model to investigate how local authorities could improve access to services and support for older people, in particular the long term impact of a new contact centre for patients. The SDM replicated the whole system for long term care, simulating the future demography and demand for care services and the nested DES model simulated the operational issues and staffing of the call centre in anticipation of growing demand for services. Zulkepli et al. [45] also used SDM-DES to model the behaviour of an integrated care system in the UK, modelling patient flow (DES) and intangible variables (SDM) related to health professionals such as motivation and stress levels.

#### Policy impact evaluation/testing

Twenty papers that utilised SDM tested the impact of policy or interventions on key health system performance or service indicators. The intended target of these policies ranged from relieving strained and under resourced healthcare services, decreasing healthcare costs to reducing patient mortality rates.

Ansah et al. [49], Brailsford et al. [50] and Desai et al. [54] aimed to reduce occupancy in acute or emergency care departments through policies that targeted elderly utilisation of these services. While demand for LTC services is expected to exponentially increase in Singapore, focus has been placed on expanding the acute care sector. Ansah et al. [49] simulated various LTC service expansion policies (static ‘current’ policy, slow adjustment, quick adjustment, proactive adjustment) and identified that proactive expansion of LTC services stemmed the number of acute care visits by elderly patients over time and required only a modest increase in the number of health professionals when compared with other policies. In Brailsford et al. [50] simulation of the entire emergency care system for Nottingham, England, policy testing indicated that while the emergency care system is operating near full capacity, yearly total occupancy of hospital beds could be reduced by re-directing emergency admissions from patients over 60 years of age (who make up around half of all admissions) to more appropriate services, such as those offered by community care facilities. To explore challenges that accompany providing care for an ageing population subject to budget restraints, Desai et al. [54] simulated the delivery and demand for social care services in Hampshire over a projected 5 year period. In offering care packages to only critical need clients and encouraging extra care services at home rather than offering residential care, the number of patients accessing acute care services reduced over the observed period.

Desai et al. [54], in addition to Taylor et al. [33] and Walker et al. [61], also examined policies that could reduce the total cost of care. Increasing the proportion of hired unqualified care workers (over qualified care workers who are employed at a higher cost rate) resulted in savings which could be fed back into care funding, although Desai et al. [54] remarked on the legal and practical limitations to this policy. Taylor et al. [33] examined the impact of shifting cardiac catheterization services from tertiary to secondary level hospitals for low risk investigations and explored how improvements could be made to services. Significant and stable improvements in service, including reduced waiting list and overall cost of service, were achieved with the implementation of strict (appropriate referral) guidelines for admitting patients. Walker et al. [61] modelled patient flow from feeder hospitals to a single sub-acute extended care facility in Victoria, Australia, to assess the impact of local rules used by the medical registrar for admission. The local admission policy which prioritised admissions from patients under the care of private doctors pushed the total cost of care over the facility budget by 6% whereas employing no prioritisation rule reduced the total cost of care to 3% under budget.

Semwanga et al. [60], Mahmoudian-Dehkordi et al. [58] and Worni et al. [63] evaluated the impact of health policy on undesirable patient outcomes (mortality and post-treatment complication rates). Semwanga et al. [60] tested the effectiveness of policies designed to promote maternal and neonatal care in Uganda, established from the literature. Policies that enabled service uptake, such as community health education, free delivery kits and motorcycle coupons were significant in reducing neonatal death over the simulated period. Mahmoudian-Dehkordi et al. [58] explored the intended and unintended consequences of intensive care unit resource and bed management policies on system performance indicators, including patient mortality. During a simulated crisis scenario, prioritising intensive care unit patient admission to general wards over emergency admissions was found to be the most effective policy in reducing total hospital mortality. Worni et al. [63] estimated the impact of a policy to reduce venous thromboembolism rates post-total knee arthroplasty surgery and identified unintentional consequences of the strategy. The policy prevented the reimbursement of patient care fees in the event that a patient was not taking the recommended prophylaxis medication and consequently develops venous thromboembolism. Simulation results indicated a positive 3-fold decrease in venous thromboembolism rates but an unintended 6-fold increase in the number of patients who develop bleeding complications as a result of compulsory prophylaxis treatment.

#### Validation (including sensitivity analysis)

Statistically-based models are usually used in quantitative data rich environments where model parameters are estimated through maximum likelihood or least-squares estimation methods. Bayesian methods can also be used to compare alternative statistical model structures. SDMs and ABMs on the other hand are not fitted to data observations in the traditional statistical sense. The data are used to inform model development. Both quantitative data and qualitative data (e.g. from interviews) can be used to inform the structure of the model and the parameters of the model. Furthermore, model structure and parameter values can also be elicited from expert opinion. This means that the nature of validation of ABMs and SDMs requires more scrutiny than that of other types of models.

With increasing complexity of such models, and to strengthen confidence in their use particularly for decision support, models are often subjected to sensitivity analysis and validation tests. Twenty-two papers that utilised SDM undertook model validation, the majority having performed behavioural validity tests (see Additional file 2 for details of validation methods for each model). Key model output such as bed occupancy [36, 50], department length of stay [62] and number of department discharges [31] were compared with real system performance data from hospitals [32, 33, 36, 48, 50, 54, 58, 59, 61, 62], local councils [54], nationally reported figs [31, 64]. as well being reviewed by experts [57, 60] as realistic. Others performed more structure orientated validity tests. Model conception [28, 60], development [30, 36, 50, 53, 54, 57, 62] and formulation [54, 56, 59] were validated by a variety of experts including health professionals [47, 53, 54, 57, 59, 62], community groups [56] and leaders [60], steering committees [36], hospital and care representatives [50, 56, 59], patient groups [60] and healthcare policy makers [60]. Further tests for structural validity included checking model behaviour when subjected to extreme conditions or extreme values of parameters [30, 31, 52, 57, 59, 60, 64], model dimensional consistency [31, 52, 57, 59, 60], model boundary adequacy [31] and mass balance [54] and integration error checks [31, 52]. Sensitivity analysis was performed to assess how sensitive model output was to changes in key parameters [49, 51, 57, 60, 64], to test the impact of parameters that had been based on expert opinion on model output [28] and varying key system parameters to test the robustness and effectiveness of policies [28, 30, 52, 53, 58] (on the assumption of imperfect policy implementation [28]).

#### Limitations of research

Most of the model limitations reported were concerned with missing parameters, feedback or inability to simulate all possible future health system innovations. Mielczarek et al. [44], Cepoiu-Martin et al. [51], Ansah et al. [49] and Rashwan et al. [31] did not take into account how future improvements in technology or service delivery may have impacted results, such as the possibility of new treatment improving patient health outcomes [51] and how this could impact the future utilisation of acute care services [49]. Walker et al. [61] and Alonge et al. [30] described how the models may not simulate all possible actions or interactions that occurred in the real system, such as all proactive actions taken by hospital managers to achieve budget targets [61] or all unintended consequences of a policy on the system [30]. De Andrade et al. [53] and Rashwan et al. [31] discussed the reality of model boundaries, that SDMs cannot encapsulate all health sub-sector behaviour and spill-over effects. Although these have been listed here as limitations, not accounting for possible future improvements in healthcare service or not simulating all possible actions in the modelled system did not prevent authors from fulfilling study objectives. When developing a SDM, it is not possible to account for all possible spill-over effects to other healthcare departments and this should not be attempted; model boundaries are set to only include variables and feedback that are pertinent to exploring the defined problem.

Simplification of model parameters was another common limitation. Wong et al. [62] stated that this would result in some model behaviour not holding in the real system, such as using weekly hospital admission and discharge averages in place of hourly rates due to the hospital recording aggregated data. This aggregation of model parameters may not have reflected real system complexity; Eleyan et al. [55] did not differentiate between service level and type of hospital when modelling health care waste production (described as future work) and Worni et al. [63] refrained from stratifying post-surgery complications by severity, potentially combining lethal and less harmful complications within the same stock (although this did not detract from the study conclusion that the rate of complications would increase as a result of the tested policy).

Data availability, lack of costing analysis and short time horizons were also considered credible limitations. Models that had been calibrated with real data were at risk of using datasets that contained measurement errors or incomplete datasets lacking information required to inform model structure or feedback [32]. Routine facility data required for model conception and formulation was unavailable which restricted the replication of facility behaviour in the model [36] and restricted validation of model behaviour [59], although it should be noted that this is only one method among many for SDM validation and the author was able to use other sources of data for this purpose. Lack of costing or cost effectiveness analysis when testing policies [60], particularly policies that required significant investment or capacity expansion [58], limited discussion on their feasibility in the real system. Models that simulated events over short time scales did not evaluate long term patient outcomes [33] or the long term effects of facility policies on certain groups of patient [57].

### ABM use in health system research (including hybrid ABM-DES)

#### Rationale for using model

The model’s ability to closely replicate human behaviour that exists in the real system was frequently cited [20–22, 25, 66], providing a deeper understanding of multiple agent decision-making [23, 67], agent networks [25] and interactions [21, 22]. The modelling method was described as providing a flexible framework capable of conveying intricate system structures [20], where simulations captured agent capacity for learning and adaptive behaviour [20, 25] and could incorporate stochastic processes that mimicked agent transition between states [25]. ABM took advantage of key individual level agent data [25] and integrated information from various sources including demographic, epidemiological and health service data [65]. The visualisation of systems and interface available with ABM software packages facilitated stakeholder understanding of how tested policies could impact financial and patient health outcomes [23], particularly those experts in the health industry with minimal modelling experience [67].

Integrating DES and ABM within a single model ensured an intelligent and flexible approach for simulating complex systems, such as the outpatient clinic described in Kittipittayakorn et al. [24]. The hybrid model captured both orthopaedic patient flow and agent decision-making that enabled identification of health care bottlenecks and optimum resource allocation [24].

#### Healthcare setting

Seven papers that utilised ABM modelled systems that were either concerned with delivering emergency or acute care^2^, ACOs or health insurance reimbursement schemes.

Liu et al. [21] and Yousefi et al. [22] modelled behaviour in EDs in Spanish and Brazilian tertiary hospitals. Liu et al. [21] simulated the behaviour of eleven key agents in the ED including patients, admission staff, doctors, triage nurses and auxiliary staff. Patients were admitted to the ED and triaged before tests were requested and a diagnosis issued. Over time, agent states changed based on their interaction with other agents such as when a doctor decided upon a course of action for a patient (sending the patient home, to another ward, or continue with diagnosis and treatment). For further details of agent type and model rules for each paper, see Additional file 3.

Yousefi et al. [22] modelled the activities of patients, doctors, nurses and receptionists in a ED. Agents could communicate with each other, to a group of other agents or could send a message to an area of the ED where other agents reside. They made decisions based on these interactions and the information available to them at the time. The main focus of the simulation was on patients who left the ED without being seen by a physician; patients decided whether to leave the ED based on a ‘tolerance’ time extracted from the literature, which changed based on their interaction with other agents. In an additional paper, Yousefi et al. [67] simulated decision-making by patients, doctors, nurses and lab technicians within a generic ED informed from the literature. Group decision-making was employed, whereby facility staff could interact with each other and reach a common solution for improving the efficacy of the department such as re-allocating staff where needed. Yousefi et al. [67], Yousefi et al. [22] and Liu et al. [21] each used a finite state machine (a computational model which describes an entity that can be in one of a finite number of states) to model interactions between agents and their states.

Liu et al. [25] and Alibrahim et al. [23] modelled the behaviour of patients, health providers and payers using series of conditional probabilities, where health providers had participated in an ACO in the United States. Liu et al. [25] presented a model where health providers within an ACO network worked together to reduce congestive heart failure patient healthcare costs and were consequently rewarded a portion of the savings from the payer agent (hypothetically, the Centers for Medicare and Medicaid Services). Patients were Medicare beneficiaries over the age of 65 who developed diabetes, hypertension and/or congestive heart failure and sought care within the network of health providers formed of three hospitals and 15 primary care physician clinics. Alibrahim et al. [23] adapted Liu et al. [25] ACO network model to allow patients to bypass their nearest medical provider in favour of an alternative provider. The decision for a patient to bypass their nearest health centre was influenced by patient characteristics, provider characteristics and the geographical distance between health providers. Providers were also given a choice on whether to participate in an ACO network, where they would then need to implement a comprehensive congestive heart failure disease management programme.

Einzinger et al. [65] created a tool that could be used to compare different health insurance reimbursement schemes in the Austrian health sector. The ABM utilised anonymous routine data from practically all persons with health insurance in Austria, pertaining to medical services accessed in the outpatient sector. In the simulation, patients developed a chronic medical issue (such as coronary heart disease) that required medical care and led to the patient conducting a search of medical providers through the health market. The patient then accessed care at their chosen provider where the reimbursement system, notified of the event via a generic interface, reimbursed the medical provider for patients care.

#### Policy impact evaluation/testing

Nine papers tested the impact of policy on key health system performance or service indicators. The intended target of these policies ranged from decreasing patient length of stay, to reducing the number of patients who leave without being seen by a physician to reducing patient mortality and hospitalisation rates.

Huynh et al. [20], Yousefi et al. [22], Yousefi et al. [67] and Kittipittayakorn et al. [24] tested policies to reduce the time agents spent performing tasks, waiting for a service or residing in parts of the system. Huynh et al. [20] modelled the medication administration workflow for registered nurses at an anonymous medical centre in the United States and simulated changes to the workflow to improve medication administration safety. Two policies were tested; establishing a rigid order for tasks to be performed and for registered nurses to perform tasks in the most frequently observed order (observed in a real medical centre) to see if this improved the average amount of time spent on tasks. Yousefi et al. [67] modelled the effects of group decision-making in ED compared with the standard approach for resource allocation (where a single supervisor allocates resources) to assess which policy resulted in improved ED performance. Turning ‘on’ group decision-making and starting the simulation with a higher number of triage staff and receptionists resulted in the largest reduction of average patient length of stay and number of patients who left without being seen. This last performance indicator was the subject of an additional paper [22], with focus on patient-to-patient interactions and how this impacted their decision to leave the ED before being seen by a physician. Four policies adapted from case studies were simulated to reduce the number of patients leaving the ED without being seen and average patient length of stay. The policy of fast-tracking patients who were not acutely unwell during triage performed well as opposed to baseline, where acutely ill patients were always given priority. Kittipittayakorn et al. [24] used ABM-DES to identify optimal scheduling for appointments in an orthopaedic outpatient clinic, with average patient waiting time falling by 32% under the tested policy.

Liu et al. [25], Alibrahim et al. [23] and Yousefi et al. [67] tested the impact of health policy on undesirable patient outcomes (patient mortality and hospitalisation rates). Liu et al. [25] modelled health care providers who operated within an ACO network and outside of the network and compared patient outcomes. Providers who operated within the ACO network worked together to reduce congestive heart failure patient healthcare costs and were then rewarded with a portion of the savings. As part of their membership, providers implemented evidence-based ﻿interventions for patients, including comprehensive discharge planning with post-discharge follow-up; this intervention was identified in the literature as key to reducing congestive heart failure patient hospitalisation and mortality, leading to a reduction in patient care fees without compromising the quality of care. The ACO network performed well, with a 10% reduction observed in hospitalisation compared with the standard care network. In another study [23] six scenarios were simulated with combinations of patient bypass capability (turned “on” or “off”) and provider participation in the ACO network (no ACO present, optional participation in ACO or compulsory participation in ACO). Provider participation in the ACO, in agreement with Liu et al. [25], led to reduced mortality and congestive heart failure patient hospitalisation, with patient bypass capability marginally increasing provider ACO participation. Yousefi et al. [67] also modelled the impact of group decision-making in ED on the number of patient deaths and number of wrong discharges i.e. patients sent to the wrong sector for care after triage and are then discharged before receiving correct treatment.

#### Validation (including sensitivity analysis)

Nine of the 11 papers that utilised ABM undertook model validation, consisting almost exclusively of behavioural validity tests. Model output, such as patient length of stay and mortality rates, was reviewed by health professionals [46, 66] and compared with data extracted from pilot studies [20], health facilities (historical) [22, 24, 46, 65, 66], national health surveys [65] and relevant literature [23, 25]. Papers presented the results of tests to determine the equivalence of variance [20] and difference in mean [20, 24] between model output and real data. Structural validity tests included extreme condition testing [23, 46] and engaging health care experts to ensure the accuracy of model framework [22, 47]. Sensitivity analysis was performed to determine how variations or uncertainty in key parameters (particularly where they had not been derived from historical or care data [65]) affected model outcomes [23, 25].

#### Limitations of research

The majority of model limitations reported were concerned the use or availability of real system or case data. Huynh et al. [20], Yousefi et al. [67] and Liu et al. [25] formulated their models using data that was obtainable, such as limited sample data extracted from a pilot study [20], national average trends [25] and data from previous studies [67]. Yousefi et al. [22] case study dataset did not contain key system feedback, such as the tolerance time of patients waiting to be seen by a physician in the ED, although authors were able to extract this data from a comparable study identified in the literature.

Missing model feedback or parameters, strict model boundaries and simplification of system elements were also considered limitations. Huynh et al. [20], Hutzschenreuter et al. [66] and Einzinger et al. [65] did not model all the realistic complexities of their system, such as all possible interruptions to tasks that occur in patient care units [20], patient satisfaction of admission processes [66] (which will be addressed in future work), how treatment influences the course of disease or that morbid patients are at higher risk of developing co-morbidity than healthier patients, which would affect the service needs and consumption needs of the patient [65]. To improve the accuracy of the model, Huynh et al. stated that further research is taking place to obtain real, clinical data (as opposed to clinical simulation lab results) to assess the impact of interruptions on workflow. Liu et al.’s [21] model boundary did not include other hospital units that may have been affected by ED behaviour and they identify this as future work, for example to include hospital wards that are affected by ED behaviour. Alibrahim et al. [23] and Einzinger et al. [65] made simplifications to the health providers and networks that were modelled, such as assuming equal geographical distances and identical care services between health providers in observed networks [23], limiting the number of factors that influenced a patients decision to bypass their nearest health provider [65] and not simulating changes to health provider behaviour based on service utilisation or reimbursement scheme in place [23]. Alibrahim et al. [23] noted that although the model was constrained by such assumptions, the focus of future work would be to improve the capability of the model to accurately study the impact of patient choice on economic, health and health provider outcomes.

### SDM-ABM use in health system research

A single paper used hybrid SDM-ABM to model health system behaviour. Djanatliev et al. [47] developed a tool that could be used to assess the impact of new health technology on performance indicators such as patient health and projected cost of care. A modelling method that could reproduce detailed, high granularity system elements in addition to abstract, aggregate health system variables was sought and a hybrid SDM-ABM was selected. The tool nested an agent-based human decision-making module (regarding healthcare choices) within a system dynamics environment, simulating macro-level behaviour such as health care financing and population dynamics. A case study was presented to show the potential impact of Mobile Stroke Units (MSU) on patient morbidity in Berlin, where stroke diagnosis and therapy could be initiated quickly as opposed to standard care. The model structure was deemed credible after evaluation by experts, including doctors and health economists.

### Comparison of SDM and ABM papers

The similarities and differences among the SDM and ABM body of literature are described in this section and shown in Table 3. A high proportion of papers across both modelling methods simulated systems that were concerned with emergency or acute care. A high number of SDM papers (11/28) simulated patient flow and pathways through emergency care [28, 31, 36, 45, 47, 50, 56–58, 61, 62] with a subset evaluating the impact of policies that relieved pressure on at capacity ED’s [28, 36, 50, 58, 62]. ABM papers simulated micro-level behaviour associated with emergency care, such as health professional and patient behaviour in EDs and what impact agent interactions have on actions taken over time [21, 22, 47, 67]. ACOs and health insurance reimbursement schemes, a common modelled healthcare setting among the ABM papers [23, 25, 65] was the focus of a single SDM paper [63] while health care waste management, a popular healthcare setting for SDM application [37, 48, 52, 55] was entirely absent among the selected ABM literature. SDM and ABM were both used to test the impact of policy on undesirable patient outcomes, including patient mortality [23, 25, 58, 60, 67] and hospitalisation rates [23, 25]. Interventions for reducing patient waiting time for services [24, 33, 53, 61, 67] and patient length of stay [22, 31, 67] were also tested using these methods, while policy exploration to reduce the total cost of care was more frequent among SDM studies [33, 54, 61].

**Table 3:** Comparison of content between SDM, ABM and hybrid models of health systems literature

	SDM papers	ABM papers	Hybrid papers
Purpose of research	*Testing policies or interventions:* • to relieve at-capacity healthcare services, reduce ward occupancy and patient length of stay [28, 31, 36, 43, 49, 50, 54, 58, 62]. • to reduce time to patient admission and treatment [33, 53, 61] • to reduce delayed discharges [31] • to increase the uptake of healthcare services and level of healthcare provision [60] • to target undesirable patient health outcomes (morbidity, mortality, post-treatment complications) [47, 58, 60, 63]. • to optimise performance-based incentive policies against health professional productivity, quality of care and volume of services [30, 59]. • to reduce the total cost of care [33, 47, 58, 60, 61, 63]. • to reduce deficit of health professionals [51] • to reduce generation of incineration-only health care waste [52] • to increase the number of patients who currently do not seek medical care [64] *Other:* • explore factors leading to undesirable emergency care system behaviour [56, 57] • simulating hospital waste management systems and predicting future waste generation [37, 48, 55]. • estimating future demand for cardiac care [44]. • exploring the impact of patient admission on health professionals stress level in an integrated care system (IC) [45]. • exploring variation in physician decision-making [32].	*Testing policies or interventions:* • to decrease the time agents spent performing tasks, waiting for a service or residing in parts of the system [20, 22, 24, 67]. • to reduce undesirable patient outcomes (mortality and hospitalisation) [23, 25, 47, 67]. • to reduce the number of patients who left a health facility without being seen by a physician [22, 67]. • to reduce number of patients who are wrongly discharged [67] • to optimise utility of resources (staff, beds) [46, 66, 67]. • on bypass rate of patients accessing care at alternative facilities [23] • to reduce total cost of care [25] *Other:* • Create tools capable of comparing health insurance reimbursement schemes [65]. • Assessing risk, allocation of resources and identifying weaknesses in emergency care services [21].	*Testing policies or interventions:* *SDM-DES* • to improve access to social support and care services [43]. *ABM-DES* • to decrease patient waiting time to be seen by a physician [24]. • to improve patient flow and length of stay through the system by optimising resource allocation [46]. *SDM-ABM* • to reduce undesirable patient outcomes (morbidity) [47]. *Other:* *SDM-DES* • Estimate the future demand for health care from patients with cardiac disease [44]. • Model patient flow through an integrated care system to estimate impact of patient admission on health care professional’s wellbeing [45].
Healthcare setting modelled	• Cardiology care [33, 53] • Elderly care or LTC services [28, 31, 36, 49–51, 54, 61, 62] • Emergency or acute care [28, 31, 36, 50, 56–58, 61, 62] • Hospital waste management [37, 48, 52, 55] • ACO or health insurance schemes [63] • MNCH [32, 60] • Orthopaedic care [63]	• Cardiology care [66] • Emergency or acute care [21, 22, 67] • ACO or health insurance schemes [23, 25, 65]	*SDM-DES* • Cardiology care [44] • Elderly care or LTC services [43–45] • Emergency or acute care [45] *ABM-DES* • MNCH [46] • Orthopaedic care [24] *SDM-ABM* • Emergency or acute care [47]
Rationale for using model	• Gain holistic perspective of system to investigate delays and bottlenecks in health facility processes, exploring counter-intuitive behaviour and monitoring interconnected processes between sub-systems over time [28, 30, 31, 36, 37, 48, 56, 58]. • Useful tool for predicting future health system behaviour and demand for care services, essential for health resource and capacity planning [48, 60]. • Configuration of model was not limited by data availability [28, 52, 64] and could integrate data from various sources when required [51]. • Used as a tool for health policy exploration and optimising health system interventions [33, 36, 51, 54, 57, 58, 64]. • Useful for establishing clinical and financial ramifications on multiple groups (such as patients and health care providers) [63]. • Identifying and simulating feedback, policy resistance or unintended system consequences [59, 61]. • Quantifying the impact of change to the health system before real world implementation [62]. • Visual learning environment enabled engagement with stakeholders necessary for model conception and validation [48, 50, 55, 57]. • Utilised by decision makers to develop and test alternative policies in a ‘real-world’ framework [31, 49, 58, 61]. • Suitable for quantitative analyses [53]. • Fast running simulation [54].	• Ability to closely replicate human behaviour that exists in the real system [20–22, 25, 66]. • Provides deeper understanding of multiple agent decision-making [23, 67], agent networks [25] and interactions [21, 22]. • Provides flexible framework capable of conveying intricate system structures [20], where simulations captured agent capacity for learning and adaptive behaviour [20, 25]. • Could incorporate stochastic processes that mimicked agent transition between states [25]. • Took advantage of key individual level agent data [25] and integrated information from various sources [65]. • Simulation allows patients to have multiple medical problems at the same time [65]. • Model can be made generalisable to other settings [65]. • Visualization of system facilitated stakeholder understanding of tested policy impact [23], particularly those in the health industry with minimal modelling experience [67].	*SDM-DES* • Enabled retention of deterministic and stochastic system variability and preservation of unique and valuable features of both methods [44]. • Capable of simulating flow of entities through system and provides rapid insight without need for large data collection [43]. • Can simulate individual variability and detailed interactions that influence system behaviour [43]. • Offered dual model functionality [44] vital for simulating human-centric activity [45], reducing the practical limitations that come with using a single simulation method to model health systems [45]. *ABM-DES* • Captured both patient flow through system and agent decision-making that enabled identification of health care bottlenecks and optimum resource allocation [24]. *SDM-ABM* • Could reproduce detailed, high granularity system elements in addition to abstract, aggregate health system variables [47].
Methods of validation	*Behavioural validity tests:* • Model output reviewed by experts [57, 60]. • Model output compared with historical data and relevant literature [31–33, 36, 48, 50, 54, 58, 59, 61, 62, 64]. *Structural validity tests:* • Model conception [28, 60], development [30, 36, 50, 53, 54, 57, 62] and formulation [54, 56, 59] validated by experts. • Extreme condition or value testing [30, 31, 52, 57, 59, 60, 64]. • Dimensional consistency checks [31, 52, 57, 59, 60]. • Model boundary accuracy checks [31]. • Mass balance checks [54]. • Integration error checks [31, 52]. *Sensitivity analysis* • to assess how sensitive model output was to changes in key parameters [49, 51, 57, 60, 64]. • to test the impact of parameters that had been based on expert opinion on model output [28]. • to test the robustness and effectiveness of policies [28, 30, 52, 53, 58, 63] (on the assumption of imperfect policy implementation [28]).	*Behavioural validity tests:* • Model output reviewed by experts [46, 66]. • Model output compared with historical data and relevant literature [20, 22–25, 46, 65, 66]. • F-test [20] and T-test [20, 24] (equivalence of variance and difference in mean tests). *Structural validity tests:* • Extreme condition or value testing [23, 46]. • Model framework reviewed by experts [22, 47]. *Sensitivity analysis:* • to determine how variations or uncertainty in key parameters (particularly where they had not been derived from historical or care data [65]) affected model outcomes [23, 25].	*Behavioural validity tests:* *ABM-DES* • Model output reviewed by experts [46]. • Model output compared with historical data [24, 46]. • T-test (difference in mean tests) [24]. *Structural validity tests:* *ABM-DES* • Extreme condition or value testing [46]. *SDM-ABM* • Model framework reviewed by experts [47]. *Sensitivity analysis:* *SDM-DES* • To assess how sensitive model output was to changes in key parameters [44].
Study limitations	• Did not consider how future improvements in technology or service delivery may impact results [31, 44, 49, 51]. • May not have simulated all possible actions or interactions that occurred in real system [30, 61]. • Model cannot encapsulate all health sub-sector behaviour and spill-over effects [31, 53]. • Simplification of real system in model [55, 62, 63]. • Lack of facility data required for model conception, formulation and validation [32, 36, 59]. • Lack of costing or cost effectiveness analysis [58, 60]. • Simulation was over a short time scale and did not evaluate long term patient outcomes [33, 57]. • Assumptions made in model development may not be generalisable to other settings [36, 63]. • Discussion with stakeholders that contributed to model development was not performed systematically [51]. • Quantifying model uncertainty was limited [64].	• Model parameterised with best information available, sometimes missing key data [20, 22, 25, 67]. • Did not model all real system complexity, simplifications made to agents and their attributes [20, 23, 65, 66]. • Did not consider all hospital units affected by possible spill-over effects [21].	*SDM-DES* • Did not consider how future improvements in technology may impact results [44]. • Did not model all real system complexity, stable number of patients with disease per age group [44]. • Lack of technology support led to simplifications in configuration of model (how information was passed between two distinct models) [45]. *ABM-DES* • Need more case studies to externally validate model [24].
Software platform	• iThink or STELLA (same software) [33, 36, 37, 48, 50, 54, 55, 57, 60, 61]. • MATLAB and Simulink [30]. • Vensim [28, 32, 52, 53, 62–64]. • Did not state [31, 49, 51, 56, 58, 59].	• AnyLogic [23, 25, 65]. • Java [66]. • Netlogo [20–22, 67].	*SDM-DES* • Vensim and Simul8 [43, 45]. • Does not state [44]. *ABM-DES* • AnyLogic [24, 46] *SDM-ABM* • AnyLogic [47].

SDM and ABM software platforms provide accessible, user-friendly visualisations of systems that enable engagement with health experts necessary for model validation [48, 50, 55, 57] and facilitate stakeholder understanding of how alternative policies can impact health system performance under a range conditions [31, 49, 58, 61]. The ability to integrate information and data from various sources was also cited as rationale for using SDM and ABM [51]. Reasons for using SDM to model health systems, as opposed to other methods, included gaining a whole-system perspective crucial for investigating undesirable or counter-intuitive system behaviour across sub-systems [28, 36, 37, 48, 56] and identifying unintended consequences or policy resistance with tested health policies [59, 61]. The ability to replicate human behaviour [20–22, 25, 66] and capacity for learning and adaptive behaviour [20, 25] was frequently cited as rationale for using ABM to simulate health systems.

Validation of SDMs and ABMs consisted mostly of behavioural validity tests where model output was reviewed by experts and compared to real system performance data or to relevant literature. Structural validity tests were uncommon among ABM papers while expert consultation on model development [30, 36, 50, 53, 54, 57, 62, 63], extreme condition [30, 31, 52, 57, 59, 60, 64] and dimensional consistency tests [31, 52, 57, 59, 60] were frequently reported in the SDM literature. The inability to simulate all actions or interactions that occur in the real system [20, 30, 61, 65, 66] and simplification of model parameters [23, 55, 62, 63, 65] were described as limitations in both SDM and ABM papers. Data availability for model conception and formulation [20, 22, 25, 32, 36, 67] and the impact of model boundaries (restricting exploration of interconnected sub-system behaviour [21, 31, 53]) were also cited limitations common to both sets of literature. Lack of costing analysis [58, 60], short time horizons [33, 57] and an inability to model future improvements in technology or service delivery [31, 44, 49, 51] were additionally cited among the SDM papers.

## Discussion

### Statement of principal findings

Our review has confirmed that there is a growing body of research demonstrating the use of SDM and ABM to model health care systems to inform policy in a range of settings. While the application of SDM has been more widespread (with 28 papers identified) there are also a growing number of ABM being used (11), just over half of which used hybrid simulation. A single paper used hybrid SDM-ABM to model health system behaviour. To our knowledge this is the first review to identify and compare the application of both SDM and ABM to model health systems. The first ABM article identified in this review was published almost a decade after the first SDM paper; this reflects to a certain extent the increasing availability of SDM and ABM dedicated software tools with the developments in ABM software lagging behind their SDM modelling counterparts.

Emergency and acute care, and elderly care and LTC services were the most frequently simulated health system setting. Both sets of services are facing exponential increases in demand with constraints on resources, presenting complex issues ideal for evaluation through simulation. Models were used to explore the impact and potential spill over effects of alternative policy options, prior to implementation, on patient outcomes, service use and efficiency under various structural and financial constraints.

### Strengths and weaknesses of the study

To ensure key papers were identified, eight databases across four research areas were screened for relevant literature. Unlike other reviews in the field [39, 40], there was no restriction placed on publication date. The framework for this review was built to provide a general overview of the SDM and ABM of healthcare literature, capturing papers excluded in other published reviews as a result of strict inclusion criteria. These include reviews that have focussed specifically on compiling examples of modelled health policy application in the literature [35] or have searched for papers with a particular health system setting, such as those that solely simulate the behaviour of emergency departments [34]. One particularly comprehensive review of the literature had excluded papers that simulated hospital systems, which we have explicitly included as part of our search framework [39].

The papers presented in this review, with selection restricted by search criteria, provide a broad picture of the current health system modelling landscape. The focus of this review was to identify models of facility-based healthcare, purposely excluding literature where the primary focus is on modelling disease progression, disease transmission or physiological disorders which can be found in other reviews such as Chang et al. [39] and Long et al. [41]. The data sources or details of how data was used to conceptualise and formulate models are not presented in this paper; this could on its own be the focus of another study and we hope to publish these results as future work. This information would be useful for researchers who want to gain an understanding of the type and format of data used to model health systems and best practice for developing and validating such models.

Literature that was not reported in English was excluded from the review which may have resulted in a small proportion of relevant papers being missed. Papers that described DES models, the other popular modelling method for simulating health system processes, were not included in this review (unless DES methods are presented as part of a hybrid model integrated with SDM or ABM) but have been compiled elsewhere [68–70]. Finally, the quality of the papers was not assessed.

### Implications for future research

A nominal number of SDM papers (9/28), an even lower proportion of ABM papers (2/11) and none of the hybrid methods papers simulated health systems based in low- or middle-income countries (LMICs). The lower number of counterpart models in LMICs can be attributed to a lack of capacity in modelling methods and perhaps the perceived scarcity of suitable data; however, the rich quantitative and qualitative primary data collected in these countries for other types of evaluation could be used to develop such models. Building capacity for using these modelling methods in LMICs should be a priority and generating knowledge of how and which secondary data to use in these settings for this purpose. In this review, we observed that it is feasible to use SDM to model low-income country health systems, including those in Uganda [60] and Afghanistan [30]. The need to increase the use of these methods within LMICs is paramount; even in cases where there is an absence of sufficient data, models can be formulated for LMICs and used to inform on key data requirements through sensitivity analysis, considering the resource and healthcare delivery constraints experienced by facilities in these settings. This research is vital for our understanding of health system functioning in LMICs, and given the greater resource constraints, to allow stakeholders and researchers to assess the likely impact of policies or interventions before their costly implementation, and to shed light on optimised programme design.

Health system professionals can learn greatly from using modelling tools, such as ABM, SDM and hybrid models, developed originally in non-health disciplines to understand complex dynamic systems. Understanding the complexity of health systems therefore require collaboration between health scientists and scientists from other disciplines such as engineering, mathematics and computer science. Discussion and application of hybrid models is not a new phenomenon in other fields but their utilisation in exploring health systems is still novel; the earliest article documenting their use in this review was published in 2010 [43]. Five of the six hybrid modelling papers [43–47] were published as conference proceedings (the exception Kittipittayakorn et al. [24]), demonstrating the need to include conference articles in systematic reviews of the literature in order to capture new and evolving applications of modelling for health systems research.

The configuration and extent to which two distinct types of models are combined has been described in the literature [71–75]. The hybrid modelling papers selected in this review follow what is described as ‘hierarchical’ or ‘process environment’ model structures, the former where two distinct models pass information to each other and the latter where one model simulates system processes within the environment of another model [72]. Truly ‘integrated’ models, considered the ‘holy grail’ [43] of hybrid simulation, where elements of the system are simulated by both methods of modelling with no clear distinction, were not identified in this review and in the wider literature remain an elusive target. In a recent review of hybrid modelling in operational research only four papers were identified to have implemented truly integrated hybrid simulation and all used bespoke software, unrestricted by the current hybrid modelling environments [76].

Of the six hybrid modelling papers, only Djanatliev et al. [47] presented a model capable of both ABM and SDM simulation. The crucial macro- and micro- level activity captured in such models represent feedback in the wider, complex system while retaining the variable behaviour exhibited by those who access or deliver healthcare. With increasing software innovation and growing demand for multi-method modelling in not only in healthcare research but in the wider research community, we need to increase their application to modelling health systems and progress towards the ‘holy grail’ of hybrid modelling.

## Conclusions

We identified 28 papers using SDM methods and 11 papers using ABM methods to model health system behaviour, six of which implemented hybrid model structures with only a single paper using SDM-ABM. Emergency and acute care, and elderly care and LTC services were the most frequently simulated health system settings, modelling the impact of health policies and interventions targeting at-capacity healthcare services, patient length of stay in healthcare facilities and undesirable patient outcomes. A high proportion of articles modelled health systems in high income countries; future work should now turn to modelling healthcare settings in LMIC to support policy makers and health system researchers alike. The utilisation of hybrid models in healthcare is still relatively new but with an increasing demand to develop models that can simulate the macro- and micro-level activity exhibited by health systems, we will see an increase in their use in the future.

## Supplementary information

**Additional file 1.** Search criteria used for each database.

**Additional file 2.** Descriptive table of validation methods used in SDM and ABM literature.

**Additional file 3.** Descriptive table of ABM model rules.

## Data Availability

Data sharing is not applicable to this article as no datasets were generated or analysed during the current study.

## Abbreviations

ACO: Accountable care organisation
ABM: Agent-based model
DES: Discrete-event simulation
ED: Emergency Department
LTC: Long-term care
LMIC: Low- and middle-income countries
SDM: System dynamics model

## Footnotes

1 One of the elderly or LTC services papers also modelled emergency or acute care but it was not the primary focus and is therefore not discussed here.

2 The single SDM-ABM paper that modelled the delivery of emergency or acute care is discussed in section ‘SDM-ABM use in health system research’.

3 Six of the emergency or acute care review papers and one of the cardiology care papers also modelled elderly or LTC services but it was not the primary focus and are therefore not discussed here.
