# Supplementary material for: Mathematical modelling for health systems research: a systematic review of system dynamics and agent-based models
Source: BMC Health Serv Res. 2019 Nov 19;19:845. doi: 10.1186/s12913-019-4627-7 (PMC6862817; doi:10.1186/s12913-019-4627-7)
Supplement: Supplementary file 1 — Additional file 1. Search criteria used for each database. [file 12913_2019_4627_MOESM1_ESM.docx]

Additional file 1: Search criteria for each database

*A.1.1 Agent-based modelling (ABM) literature search strategy*

*Note:* ^1^The search was sent twice to include results for ‘agent based’ and agent-based’.

| Database | Agent-based modelling (ABM) literature search strategy |
| --- | --- |
| ACM Library | +(("agent-based" "agent based") +("model*") +("health system*" "health care" "healthcare" "health service*" "health polic*" "health facil*" "primary care" "secondary care" "tertiary care" "hospital*")) |
| Cochrane | 1. (health system*):ti,ab,kw OR (health care*):ti,ab,kw OR (healthcare):ti,ab,kw OR (health service*):ti,ab,kw OR (health polic*):ti,ab,kw OR (health facil*):ti,ab,kw OR (primary care):ti,ab,kw OR (secondary care):ti,ab,kw OR (tertiary care):ti,ab,kw OR (hospital*):ti,ab,kw 2. (agent based):ti,ab,kw OR (agent-based):ti,ab,kw 3. (model*):ti,ab,kw 4. (#1 AND #2 AND #3) |
| Econlit | 1. (health system* OR health care OR healthcare OR health service* OR health polic* OR health facil* OR primary care OR secondary care OR tertiary care OR hospital*).ab,ti,kw. 2. (agent-based OR agent based).ab,ti,kw. 3. (model*).ab,ti,kw. 4. 1 and 2 and 3 |
| Embase Classic+Embase | 1. (health system* OR health care OR healthcare OR health service* OR health polic* OR health facil* OR primary care OR secondary care OR tertiary care OR hospital*).ab,ti,kw. 2. (agent-based OR agent based).ab,ti,kw. 3. (model*).ab,ti,kw. 4. 1 and 2 and 3 |
| Global Health | 1. (health system* OR health care OR healthcare OR health service* OR health polic* OR health facil* OR primary care OR secondary care OR tertiary care OR hospital*).ab,ti. 2. (agent-based OR agent based).ab,ti. 3. (model*).ab,ti. 4. 1 and 2 and 3 |
| HMIC Health Management Information Consortium | 1. (health system* OR health care OR healthcare OR health service* OR health polic* OR health facil* OR primary care OR secondary care OR tertiary care OR hospital*).ab,ti. 2. (agent-based OR agent based).ab,ti. 3. (model*).ab,ti. 4. 1 and 2 and 3 |
| MathSciNet^1^ | "(Anywhere=(health*) AND Anywhere=(agent-based)^1^ AND Anywhere=(model*))" |
| Ovid MEDLINE(R) | 1. (health system* OR health care OR healthcare OR health service* OR health polic* OR health facil* OR primary care OR secondary care OR tertiary care OR hospital*).ab,ti,kw. 2. (agent-based OR agent based).ab,ti,kw. 3. (model*).ab,ti,kw. 4. 1 and 2 and 3 |

*A.1.2 System dynamics modelling (SDM) literature search strategy*

*Note:* ^1^The search was sent twice to include results for ‘agent based’ and agent-based’.

| Database | System dynamics modelling (SDM) literature search strategy |
| --- | --- |
| ACM Library | +(("system dynamic*") +("model*") +("health system*" "health care" "healthcare" "health service*" "health polic*" "health facil*" "primary care" "secondary care" "tertiary care" "hospital*")) |
| Cochrane | 1. (health system*):ti,ab,kw OR (health care*):ti,ab,kw OR (healthcare):ti,ab,kw OR (health service*):ti,ab,kw OR (health polic*):ti,ab,kw OR (health facil*):ti,ab,kw OR (primary care):ti,ab,kw OR (secondary care):ti,ab,kw OR (tertiary care):ti,ab,kw OR (hospital*):ti,ab,kw 2. (system dynamic*):ti,ab,kw 3. (model*):ti,ab,kw 4. (#1 AND #2 AND #3) |
| Econlit | 1. (health system* OR health care OR healthcare OR health service* OR health polic* OR health facil* OR primary care OR secondary care OR tertiary care OR hospital*).ab,ti,kw. 2. (system dynamic*).ab,ti,kw. 3. (model*).ab,ti,kw. 4. 1 and 2 and 3 |
| Embase Classic+Embase | 1. (health system* OR health care OR healthcare OR health service* OR health polic* OR health facil* OR primary care OR secondary care OR tertiary care OR hospital*).ab,ti,kw. 2. (system dynamic*).ab,ti,kw. 3. (model*).ab,ti,kw. 4. 1 and 2 and 3 |
| Global Health | 1. (health system* OR health care OR healthcare OR health service* OR health polic* OR health facil* OR primary care OR secondary care OR tertiary care OR hospital*).ab,ti,kw. 2. (system dynamic*).ab,ti,kw. 3. (model*).ab,ti,kw. 4. 1 and 2 and 3 |
| HMIC Health Management Information Consortium | 1. (health system* OR health care OR healthcare OR health service* OR health polic* OR health facil* OR primary care OR secondary care OR tertiary care OR hospital*).ab,ti. 2. (system dynamic*).ab,ti. 3. (model*).ab,ti. 4. 1 and 2 and 3 |
| MathSciNet | "(Anywhere=(health*) AND Anywhere=(system dynamic*) AND Anywhere=(model*))" |
| Ovid MEDLINE(R) | 1. (health system* OR health care OR healthcare OR health service* OR health polic* OR health facil* OR primary care OR secondary care OR tertiary care OR hospital*).ab,ti,kw. 2. (system dynamic*).ab,ti,kw. 3. (model*).ab,ti,kw. 4. 1 and 2 and 3 |
