# Supplementary material for: Mathematical modelling for health systems research: a systematic review of system dynamics and agent-based models
Source: BMC Health Serv Res. 2019 Nov 19;19:845. doi: 10.1186/s12913-019-4627-7 (PMC6862817; doi:10.1186/s12913-019-4627-7)
Supplement: Supplementary file 2 — Additional file 2. Descriptive table of validation methods used in SDM and ABM literature. [file 12913_2019_4627_MOESM2_ESM.docx]

Additional file 2: Descriptive table of validation methods used in SDM and ABM literature

*A.2.1 Validation method used and outcome variables validated for SDMs and ABMs*

| **System dynamics models (SDMs)** | | | |
| --- | --- | --- | --- |
| Paper/Year/Ref | Purpose | Validation method(s) used | Any outcome variable(s) validated? (behaviour/parameter/structure confirmation tests) |
| Al-Khatib (2016) [48] | - Assess the impact of key factors on the hospital waste management system and compare the future total waste output between private, charitable and government hospitals. | *Behaviour validity*   - Model output compared to real data. | - Unclear which model output was validated with data collected from Nablus city hospitals. |
| Alonge (2017) [30] | - Explore effective implementation structure for improving health system performance through pay-for-performance (P4P) initiative. | *Structure validity*   - Direct structure tests (structure-confirmation test, parameter-confirmation test). - Structure-orientated behavioural tests (phase relationship test, extreme-condition test, behaviour sensitivity test). | - Each element (and how they were parameterised) of the model was confirmed by healthcare and P4P experts in Afghanistan. ‘Gaming’ element of model reported in the literature. |
| Ansah (2014) [49] | - Assess the impact of different long-term care (LTC) capacity policies on uptake of acute care, demand for and utilisation of LTC services. |  |  |
| Brailsford (2004) [50] | - To determine how emergency and on demand care is currently configured and what policies could alleviate pressure on the health system. | *Behaviour validity*   - Model output compared to real data.   *Structure validity*   - Direct structure tests (dimensional consistency test, structure-confirmation test, parameter-confirmation test). | - Total daily bed occupancy and individual ward occupancy are given as examples of model output that were validated with real health facility performance data from Nottingham hospitals. - The model was developed in close collaboration with the Steering Committee (representatives from all healthcare providers in Nottingham). |
| Brailsford (2010)^*1^ [43] | - Investigate how local authorities such as Hampshire County Council (HCC) can improve access to services and support for older people, in particular assess the long-term impact of a new contact centre for patients. |  |  |
| Cepoiu‐Martin (2018) [51] | - To examine patient transition from home to supportive living (SL) or long term care (LTC) in persons with dementia and discern policy impact on the deficit of nurses and health care assistants. |  |  |
| Chaerul (2008) [37] | - To determine key factors that impact the management of hospital waste and predict future waste output. | *Behaviour validity*   - Model output compared to published data. | - Unclear which model output was validated with published data on health and waste management in Jakarta, Indonesia. |
| Ciplak (2012) [52] | - To predict future healthcare waste production and optimise the management of healthcare waste. | *Behaviour validity*   - Model output compared to published data.   *Structure validity*   - Direct structure tests (dimensional consistency). - Structure-orientated behavioural tests (extreme-condition test, integration error test). | - Population and total healthcare waste materials generated are given as examples of model output that were validated with published data on health and waste management in Istanbul, Turkey. |
| De Andrade (2014) [53] | - To examine the reasons for delayed ST-segment elevation myocardial infarction (STEMI) treatment and explore interventions that can speed up wait time in primary care facilities. | *Structure validity*   - Direct structure tests (structure confirmation test). | - The model was developed in close collaboration (‘mediated modelling’) with health professionals at a primary hospital in Foz do Iguaçu, Brazil. |
| Desai (2008) [54] | - To forecast demand for older people's services and explore the future impact of challenges that accompany an ageing population. | *Behaviour validity*   - Model output compared to real data.   *Structure validity*   - Direct structure tests (structure-confirmation test, parameter-confirmation test). - Structure-orientated behavioural tests (mass-balance check) | - Researchers worked in close collaboration and consulted staff at Hampshire County Council during model conceptualisation and formulation. - Model output (stocks) for initial contact, eligibility and initial assessment, care manager assessment and create a care package were compared with real data from Hampshire County Council. |
| Djanatliev (2012)^*2^ [47] | - Presenting the functionality of the Prospective Health Technology Assessment (ProHTA) tool, which can simulate the impact of optimised technology prospectively before physical development. | *Structure validity*   - Direct structure tests (structure-confirmation test, parameter-confirmation test). | - Model achieved credibility from review by experts (doctors, health economists, medical informaticians and knowledge management experts). |
| Eleyan (2013) [55] | - To predict general and medical waste generation for a complex hospital waste management system. | *Behaviour validity*   - Model output compared to published data. | - Model output, general waste generated and hazardous waste generated, were compared with published waste data from hospitals in Iran. |
| Esensoy (2018) [28] | - Transformation of stroke care to implement best practice. | *Behaviour validity*   - Model output shared with experts.   *Structure validity*   - Direct structure tests (structure-confirmation test, parameter-confirmation test). | - Preliminary model results were shared with policy and stroke experts for validation. - Model development conducted in close collaboration with Ministry of Health and Long-term Care and the Central East Local Integration Network in Ontario, Canada. |
| Ghaffarzad. (2013) [32] | - To explore physician decision making behind scheduled caesarean delivery (CD), unplanned CD and vaginal delivery (VD) and examine factors that influence procedure variation. | *Behaviour validity*   - Model output compared to out-of-sample real data. | - Model output, total scheduled caesarean deliveries and total caesarean deliveries, was compared with empirical data from the Florida all-payer hospital discharge database (concerning deliveries at non-federal acute-care hospitals). |
| Lane (1998) [56] | - Explore the factors that lead to delays in Accident and Emergency Departments (A&E) and to elective admissions. | *Structure validity*   - Direct structure tests (structure confirmation test, parameter confirmation test). | - Researchers worked closely with the A&E department of a London hospital, pseudonym ‘St Danes’ during model development. - Aspects of model formulation were reviewed by members of Casualty Watch, the London Ambulance Service and the Emergency Bed Service. |
| Lane (2000) [57] | - The model depicts the performance of Accident and Emergency (A&E) at acute hospitals, investigating the sensitivity of waiting times to hospital bed numbers. | *Behaviour validity*   - Model output shared with experts.   *Structure validity*   - Direct structure tests (dimensional consistency, structure confirmation test, parameter confirmation test). - Structure-orientated behavioural tests (extreme-condition test). | - Model output (shown using graphs) and performance indicators were judged as realistic by staff from (pseudonym) St Danes hospital, London, England. - Model structure was reviewed by experts (A&E system) and model parameters were confirmed using available hospital process information. - Aspects of model formulation were reviewed by members of Southwark Community Health Council, Casualty Watch, the London Ambulance Service, the Emergency Bed Service and the Registrar at St Danes. |
| Lattimer (2004) [36] | - To evaluate ‘front door’ services of local emergency and urgent care facilities and test proposals for system change. | *Behaviour validity*   - Model output compared to real data.   *Structure validity*   - Direct structure tests (structure confirmation test, parameter confirmation test). | - Model output compared to data from the emergency and urgent care system in Nottingham, England. Daily bed occupancy rates were given as an example of model output that was validated. - The project team, steering group and health professionals from Nottingham emergency and urgent care system contributed to the development and refinement of the quantitative model. |
| Mahmoudia. (2017) [58] | - To explore the intended and unintended consequences of Intensive Care Unit (ICU) resource and bed management policies on patient mortality, emergency departments (ED) and general wards. | *Behaviour validity*   - Model output compared to health facility data extracted from the literature. | - Model output, specifically average occupancy of ED, average ICU occupancy and ward occupancy, are compared to relevant health facility statistics identified in the literature. |
| Meker (2015) [59] | - To describe performance-based payment systems (PBPS) in second-step public hospitals and the impact on process measures in hospitals. | *Behaviour validity*   - Model output compared to real data.   *Structure validity*   - Direct structure tests (dimensional consistency). - Structure-orientated behavioural tests (extreme-condition test). | - Model output, specifically number of patients examined per month, number of tests performed per month and number of surgeries performed per month, are compared to data from a second-step public hospital in Istanbul. |
| Mielczarek (2016)^*1^ [44] | - To estimate the future demand for healthcare from patients with cardiac disease. |  |  |
| Rashwan (2015) [31] | - To explore the flow of elderly patients through the Irish healthcare system and anticipate the growing demand for services over the next five years. | *Behaviour validity*   - Model output compared to reported data.   *Structure validity*   - Direct structure tests (dimensional consistency, structure-confirmation test). - Structure-orientated behavioural tests (boundary adequacy, extreme-condition test, integration error test). | - The model structure was discussed and reviewed by Irish Health Service Executive (HSE) officials and domain experts. - Model output, specifically number of delayed discharges for each possible destination (home, another hospital, rehabilitation, convalescence, long term care, death, other), were compared to annual delayed discharge figures reported by the HSE. |
| Semwanga (2016) [60] | - To capture the dynamics of the Ugandan health system and evaluate what impact interventions might have on neonatal care. | *Behaviour validity*   - Model output compared to historical and reported data.   *Structure validity*   - Direct structure tests (dimensional consistency, parameter-confirmation, structure-confirmation test). - Structure-orientated behavioural tests (mass-balance test, extreme-condition test). | - Model output, including graphical behaviour and results, were compared to historical/national health surveys and reports. - During brainstorming sessions, researchers and neonatal and maternal healthcare staff from hospitals in Uganda were asked to validate the model structure. |
| Taylor (2005) [33] | - To examine the impact of shifting cardiac catheterization (CC) services from tertiary to secondary level for low risk investigations and explore how improvements could be made to services. | *Behaviour validity*   - Model output compared to historical data (Theil inequality statistic, R^2^ statistic, MAPE statistic). | - Simulated model output was compared with real data from Ribsley Hospital for CC services, specifically district-based elective CC investigation rate, elective CC investigation referral rate, average time spent on CC investigation list, CC investigation waiting list, elective CC investigation rate, tertiary-based elective CC investigation rate. |
| Walker (2003) [61] | - To model patient flow from feeder hospitals to a ﻿sub acute extended care hospital to show the impact of local rules used by the medical registrar (medical admitting officer). ﻿ | *Behaviour validity*   - Model output compared to historical data. | - Model results were compared with historical data from a sub-acute extended care facility in Australia. |
| Wong (2010) [62] | - To evaluate if smoothing the number of discharges over the week relieves the pressure on emergency departments (ED). | *Behaviour validity*   - Model output compared to historical data.   *Structure validity*   - Direct structure tests (structure-confirmation test, parameter-confirmation test). | - Model output, specifically those concerning ED and ward censuses and their respective length of stay, were compared with historical data from the Toronto General Hospital, Canada. - Staff physicians at the hospital were engaged during the study and their feedback was used to validate model structure, assumptions and feedback. |
| Worni (2012) [63] | - To estimate what impact a policy to deny reimbursement of total knee arthroplasty (TKA) patient fees will have on venous thromboembolism (VTE) rates and any unintentional consequences. |  |  |
| Yu (2015) [64] | - To explore the driving factors for a high proportion of patients in China not seeking medical care (also known as potential medical demand) and examine possible interventions. | *Behaviour validity*   - Model output compared to historical data. | - Model output, specifically number of visits to hospital, were compared to historical data from the Chinese Health Statistical Yearbook. |
| Zulkepli (2012)^*1^ [45] | - Present a case study using hybrid modelling (SDM-DES), explore patient flow in an integrated care system (IC) and the impact of patient admission on health professional stress level. |  |  |
| **Agent-based models (ABMs)** | | | |
| Alibrahim (2018) [23] | To explore the effect of patient choice on the healthcare market, specifically providers that form accountable care organisations (ACO). | *Behaviour validity*   - Model output compared to published data.   *Structure validity*   - Structure-orientated behavioural tests (extreme-condition test, parameter-confirmation tests). | - Model results relating to financial and population outcomes were compared with those from ACO studies, identified in the literature. - Patient bypass rates were comparable with previous studies of Medicare patients and per-patient payment figures were in line with estimates identified in the literature. |
| Djanatliev (2012)^*2^ [47] | Presenting the functionality of the Prospective Health Technology Assessment (ProHTA) tool, which can simulate the impact of optimised technology prospectively before physical development. | *Structure validity*   - Direct structure tests (conceptual confirmation test). | - Model achieved credibility from review by experts (doctors, health economists, medical informaticians and knowledge management experts). |
| Einzinger (2013) [65] | To create a tool capable of comparing reimbursement schemes in outpatient care. | *Behaviour validity*   - Model output compared to reported data.   *Structure validity*   - Structure-orientated behavioural tests (parameter-confirmation tests). | - The overall prevalence of disease in the model was comparable with national health survey data, confirming parameterisation of model with routine care data was valid. |
| Hutzsch. (2008) [66] | To determine which mix of patients should be admitted to specialised hospitals to optimise resource utility and to consider the impact of unplanned patient arrivals on this process. | *Behaviour validity*   - Model output compared to real data and shared with experts. | - Model output, including the annual number of surgery patients, number of admission requests and back-up capacity usage in medium care, were comparable with real data from the Catharina Hospital Eindhoven, the Netherlands. - Domain experts from the hospital were consulted and determined model output to be credible. |
| Huynh (2012) [20] | To assess the impact of redesigning medication administration process (MAP) workflow for registered nurses to improve medication administration safety. | *Behaviour validity*   - Model output compared to real data (F-test, T-test) | - Model output, specifically amount of time spent performing tasks and variation in number of tasks performed, were compared to observed pilot study data using t-tests. - An F-test was also used to determine equivalence of variance between simulated and observed pilot study data. |
| Kittipitta. (2016)^*3^ [24] | To examine patient flow in an outpatient clinic of an orthopedic department and explore interventions that can improve clinical services to reduce patient waiting times. | *Behaviour validity*   - Model output compared to real data (T-test) | - Model output, specifically average waiting time, average throughput time and average utilisation, were compared to observed and recorded department operations using t-tests. |
| Liu (2014) [21] | To develop a tool that can be used as a decision support system for managers of emergency departments (ED) to assess risk, allocation of resources and identify weakness in emergency care service. |  |  |
| Liu (2016) [25] | To explore how accountable care organisations (ACO) can impact payers, healthcare providers and patients under a shared savings payment model for congestive heart failure (CHF) and achieve optimal outcomes. | *Behaviour validity*   - Model output compared to published data. | - Model output, specifically CHF-related hospitalisation rate and mortality rate, were compared to published results from clinical trials and national health reports. |
| Viana (2018)^*3^ [46] | To examine and improve patient flow through a pregnancy outpatient clinic in light of the uncertainty in demand for services from overdue patients. | *Behaviour validity*   - Model output compared to real data and reviewed by experts.   *Structure validity*   - Structure-orientated behavioural tests (structure-confirmation test, extreme-condition test). | - Model output, specifically arrival patterns, was compared with historical data from the outpatient clinic at Akershus University Hospital, Norway and patient length of stay results were deemed credible by clinic staff. - Clinic staff also reviewed the visualisation of the model. |
| Yousefi (2017) [67] | To apply group decision-making techniques for emergency department (ED) resource allocation and determine whether this approach improves performance indicators. |  |  |
| Yousefi (2018) [22] | To examine the behaviour of patients who leave public hospital emergency departments (ED) without being seen and the impact of preventative policies. | *Behaviour validity*   - Model output compared to reported data (t-test).   *Structure validity*   - Structure-orientated behavioural tests (structure-confirmation tests). | - Model output, specifically total time patients spent in hospital and weekly number of discharged patients, was compared to historical data from the ED at Hospital Risoleta Tolentino Neves, Minas Gerais, Brazil using t-tests. - The simulation model was reviewed by the hospital manager and district co-ordinator. |

*Note: ^*1^ Articles implemented SDM-DES hybrid modelling. ^*2^ Articles implemented SDM-ABM hybrid modelling. ^*3^ Articles implemented ABM-DES hybrid modelling.*
