# Supplementary material for: Mathematical modelling for health systems research: a systematic review of system dynamics and agent-based models
Source: BMC Health Serv Res. 2019 Nov 19;19:845. doi: 10.1186/s12913-019-4627-7 (PMC6862817; doi:10.1186/s12913-019-4627-7)
Supplement: Supplementary file 3 — Additional file 3. Descriptive table of ABM model rules. [file 12913_2019_4627_MOESM3_ESM.docx]

Additional file 3: Descriptive table of ABM model rules

*A.3.1 ABM agents, model rules and source of rules*

| ***Agent-based models (ABMs)*** | | | | |
| --- | --- | --- | --- | --- |
| Paper/Year/Ref | Purpose | Type of agents | Key model rules | Type/source of rules |
| Alibrahim (2018) [23] | To explore the effect of patient choice on the healthcare market, specifically providers that form accountable care organisations (ACO). | - Patient - Provider (hospitals, primary care physician clinics) - Payer (Medicare) | - Patients could bypass their assigned healthcare provider based on patient characteristics (age, race, gender, income), provider characteristics (mortality rate, hospitalisation rate, presence of disease management programme in place by membership of ACO), and distance between providers. If patients chose to bypass, they must travel 60 mins to other facility. - Providers delivered congestive heart failure care to patients and could chose to be part of the ACO network. If providers chose to be part of the ACO network they would share cost savings with the payer (Medicare) but have to provide comprehensive disease management programs. - Payer agents calculate the savings made between providers participating in the ACO or not and return a percentage of savings to providers. | - Literature (epidemiological studies, cost-effectiveness studies, national/public health surveys). - Psychological theory (Theory of planned behaviour). - Behaviour economics. |
| Einzinger (2013) [65] | To create a tool capable of comparing reimbursement schemes in outpatient care. | - Patient - Medical provider | - A patient develops a chronic medical issue (such as coronary heart disease) that requires care, leading to the patient conducting a search of medical providers through the health market with preference for those who are closer. - The health market (classified as a single object in model) returns a list of suitable providers using distance from the patient, suitable wait time and selecting providers who can provide the highest number of services required. - The patient accesses care at their chosen provider. The reimbursement system is notified of this event via a generic interface and reimburses the medical provider for the patients care. | - Utility/game theory (standard gamble utility assessment technique). - Heuristics (greedy algorithm). - Agent behaviour adapted from a prototype, universal model of a healthcare system. |
| Djanatliev (2012)^*2^ [47] | Presenting the functionality of the Prospective Health Technology Assessment (ProHTA) tool, which can simulate the impact of optimised technology prospectively before physical development. | - Patient - Mobile Stroke Unit | - Mobile Stroke Units (MSU), a new healthcare technology innovation, are presented as a case study. - The ABM module simulates patient state (generally categorised as prevention, pre-treatment, treatment, post-treatment stages) which are reached by patients traversing a workflow, with decisions based on set probabilities when a patient has a stroke i.e. patient contacting emergency services, contacting GP, going directly to the hospital. - If a MSU is available, this is dispatched to treat the patient which would lead to reduced long term complications. | - Observational studies. - Determined by domain experts. |
| Hutzsch. (2008) [66] | To determine which mix of patients should be admitted to specialised hospitals to optimise resource utility and to consider the impact of unplanned patient arrivals on this process. | - OR scheduling agent - Resource agent | - OR scheduling agent manages the use of cardiothoracic surgery (CTS) operating room. - Resource agents are each of the units that form the CTS and intensive care unit (ICU) departments, such as the high care unit of CTS, the main ward for CTS, high care intensive care unit. - Patient priority and care pathway is determined by selected patient characteristics. - Each medical unit has their own preference for the type of patient they will admit. Hospital resources (such as use of operating rooms) are limited and with their availability in flux due to the need from other surgical disciplines and admission of emergency patients. - Where emergency patients are admitted, bottlenecks can occur in ICU where beds are needed by elective CTS patients but priority is given to acute cases. | - Routine health facility data. - Determined by healthcare experts. |
| Huynh (2012) [20] | To assess the impact of redesigning medication administration process (MAP) workflow for registered nurses to improve medication administration safety. | - Registered nurse | - A registered nurse is engaged in a single task until its completion but can be interrupted by another health professional. - Based on transition probabilities, the nurse then moves on to a new task until all tasks are completed. | - Observational study. |
| Kittipitta. (2016)^*3^ [24] | To examine patient flow in an outpatient clinic of an orthopaedic department and explore interventions that can improve clinical services to reduce patient waiting times. | - Orthopaedic outpatient - Doctor - Nurse - Healthcare assistant - Radiologist - Biomedical scientist - Administration staff - Patient information system^p^ - Examination centre^p^ - Loudspeaker system^p^ | - Patients who are 85 years or older or have a particular condition requiring a fast track consultation are attended to by a doctor first, all other patients are sent to the waiting area. - Scheduling of patients in the waiting area is dependent on the number of walk-in patients and scheduled patients are waiting. - The doctor decides if the patient should be sent for an examination (to then return for another consultation), requires medication and when they are to be sent home. - The patient collects any medication required from the pharmacy. | - Observational study. - Determined by healthcare experts. - Routine health facility data. |
| Liu (2014) [21] | To develop a tool that can be used as a decision support system for managers of emergency departments (ED) to assess risk, allocation of resources and identify weakness in emergency care service. | - Patient - Admission staff - Triage Nurse - Doctor - Auxiliary staff - Nurse - Laboratory test - Internal test - External test - Ambulance - Carebox | - Each agent has assigned behaviour such as waiting for the next task, arranging a test, providing treatment, moving a patient to a different area of the ward etc. - Patients are admitted and triaged before tests are requested and a diagnosis issued. Over time a patient’s status may change where the doctor will decide a new course of action (send the patient home, to another ward, or continue with diagnosis and treatment). | - Determined by healthcare experts. - Routine health facility data. |
| Liu (2016) [25] | To explore how accountable care organisations (ACO) can impact payers, healthcare providers and patients under a shared savings payment model for congestive heart failure (CHF) and achieve optimal outcomes. | - Patient^p^ - Provider (hospitals, primary care physician clinics) - Payer (Medicare) | - Patients were passive and were not decision-makers. - Providers considered whether to conduct the CHF intervention. Provider behaviour was dependent on the financial return of conducting the intervention, patient health outcomes, peer pressure from other providers and perceived difficulty in conducting intervention. - Payer agents calculate the savings made between providers participating in the ACO or not and return a percentage of savings to providers. | - Literature (epidemiological studies, cost-effectiveness studies, national/public health surveys). - Psychological theory (Theory of planned behaviour). - Behaviour economics. |
| Viana (2018)^*3^ [46] | To examine and improve patient flow through a pregnancy outpatient clinic in light of the uncertainty in demand for services from overdue patients. | - Patient | - The assigned characteristics of the patient (particularly if the pregnancy is considered overdue) and utilisation of staff will determine where the patient is sent after arriving for her appointment, when the patient is attended to by a midwife or doctor and how long the entire process takes. | - Determined by healthcare experts. - Routine health facility data. |
| Yousefi (2017) [67] | To apply group decision-making techniques for emergency department (ED) resource allocation and determine whether this approach improves performance indicators. | - Patient - Doctor - Technician - Triage nurse - Emergency room nurse - Receptionist | - Behaviour of agents are modelled by a finite state machine, where agent interactions result in a change of state. - Each agent has a set list of possible tasks they may complete i.e. patients can wait for treatment, then receive treatment, then move to a different section of the ED etc. - Agent communication also informed group decision-making whereby a group of agents could decide where to place resources (allocate a nurse to a different area of the ED) if an area of the ED was struggling. | - Observational study. - Multi-attribute decision making theory. - Literature (modelling studies, observational studies, routine health facility data). |
| Yousefi (2018) [22] | To examine the behaviour of patients who leave public hospital emergency departments (ED) without being seen and the impact of preventative policies. | - Patient - Doctor - Nurse - Receptionist | - Agents can communicate with each other, to a group of agents or agents can send a message to an area of the ED where other agents reside. - Agents make decisions based on these interactions and information available to them at the time. - Patients decide whether to leave the emergency department based on a tolerance time, which can change upon interaction with other agents. | - Cellular automata. - Observational study. - Literature (modelling studies, observational studies, routine health facility data). - Determined by healthcare experts. - Routine health facility data. |

*Note: ^*2^ Articles implemented SDM-ABM hybrid modelling. ^*3^ Articles implemented ABM-DES hybrid modelling. ^p^ Considered in the published model as a passive, non-decision-making agent.*
